# Transitional CXCL14^+^ cancer‐associated fibroblasts enhance tumour metastasis and confer resistance to EGFR‐TKIs, revealing therapeutic vulnerability to filgotinib in lung adenocarcinoma

**DOI:** 10.1002/ctm2.70281

**Published:** 2025-03-31

**Authors:** Weijiao Xu, Haitang Yang, Ke Xu, Anshun Zhu, Sean R. R. Hall, Yunxuan Jia, Baicheng Zhao, Enshuo Zhang, Gang Liu, Jianlin Xu, Thomas M. Marti, Ren‐Wang Peng, Patrick Dorn, Yongliang Niu, Xufeng Pan, Yajuan Zhang, Feng Yao

**Affiliations:** ^1^ Department of Thoracic Surgery Shanghai Chest Hospital, Shanghai Jiao Tong University School of Medicine Shanghai China; ^2^ Department of Thoracic Surgery, First Affiliated Hospital of Wenzhou Medical University Wenzhou Medical University Wenzhou China; ^3^ Department of General Thoracic Surgery Inselspital, Bern University Hospital Bern Switzerland; ^4^ Department of BioMedical Research (DBMR) University of Bern Bern Switzerland; ^5^ Department of Respiratory Medicine Shanghai Chest Hospital, Shanghai Jiao Tong University Shanghai China; ^6^ Department of Respiratory and Critical Care Medicine No. 2 People`s Hospital of Fuyang City, Fuyang Infectious Disease Clinical College of Anhui Medical University Fuyang China; ^7^ Shanghai Institute of Thoracic Oncology Shanghai Chest Hospital, Shanghai Jiao Tong University School of Medicine Shanghai China; ^8^ Present address: Iovance Biotherapeutics, Inc. San Carlos CA USA

**Keywords:** cancer‐associated fibroblasts, EGFR‐TKIs, heterogeneity, lung adenocarcinoma, metastasis, survival

## Abstract

**Background:**

The heterogeneity of cancer‐associated fibroblasts (CAFs) has become a crucial focus in understanding cancer biology and treatment response, revealing distinct subpopulations with specific roles in tumor pathobiology. CAFs have also been shown to promote resistance in lung cancer cells to epidermal growth factor receptor‐tyrosine kinase inhibitors (EGFR‐TKIs). However, the specific CAF subsets responsible for driving tumor advancement and resistance to EGFR‐TKIs in lung adenocarcinoma (LUAD) remain poorly understood.

**Methods:**

We integrate multiple scRNA‐seq datasets to identify cell subclusters most relevant to tumor stage, patient survival, and EGFR–TKIs response. Additionally, in vitro and in vivo experiments, clinical tissue sample immunohistochemistry and patient plasma ELISA experiments are performed to validate key findings in independent LUAD cohorts.

**Results:**

By analyzing multiple scRNA‐seq and spatial transcriptomic datasets, we identified a unique subset of CXCL14+ myofibroblastic CAFs (myCAFs), emerging during the early differentiation phase of pan‐cancer invasiveness‐associated THBS2⁺ POSTN⁺ COL11A1⁺ myCAFs. Notably, plasma levels of CXCL14 in LUAD patients correlate significantly with tumor stage. Mechanistically, this subset enhances tumor aggressiveness through epithelial‐to‐mesenchymal transition and angiogenesis. Among standard treatment regimens, transitional CXCL14+ myCAFs specifically confer resistance to EGFR‐TKIs, while showing no significant impact on chemotherapy or immunotherapy outcomes. Through a pharmacological screen of FDA‐approved drugs, we identified Filgotinib as an effective agent to counteract the EGFR‐TKIs resistance conferred by this CAF subset.

**Conclusions:**

In summary, our study highlights the role of the differentiated stage from transitional CXCL14+ myCAFs to invasiveness‐associated myCAFs in driving tumor progression and therapy resistance in LUAD, positioning Filgotinib as a promising targeted therapy for this process. These insights may enhance patient stratification and inform precision treatment strategies in LUAD.

**Key points:**

- Single‐cell analysis identifies transitional CXCL14^+^ myofibroblastic cancer‐associated fibroblasts (myCAFs) predominantly exist in the advanced‐stage lung adenocarcinoma (LUAD).
- Transitional CXCL14^+^ myCAFs fuel metastasis by promoting epithelial–mesenchymal transition (EMT) and angiogenesis on the spatial level.
- CXCL14 is a potential diagnostic marker for LUAD patients and predict the occurrence of metastasis.
- Transitional CXCL14^+^ myCAFs induce the resistance to epidermal growth factor receptor‐tyrosine kinase inhibitors (EGFR‐TKIs) and JAK1 inhibitor, filgotinib could reverse the effect.

## Graphical abstract

1. Single‐cell analysis identifies transitional CXCL14^+^ myofibroblastic cancer‐associated fibroblasts (myCAFs) predominantly exist in the advanced‐stage lung adenocarcinoma (LUAD).
2. Transitional CXCL14^+^ myCAFs fuel metastasis by promoting epithelial–mesenchymal transition (EMT) and angiogenesis on the spatial level.
3. CXCL14 is a potential diagnostic marker for LUAD patients and predict the occurrence of metastasis.
4. Transitional CXCL14^+^ myCAFs induce the resistance to epidermal growth factor receptor‐tyrosine kinase inhibitors (EGFR‐TKIs) and JAK1 inhibitor, filgotinib could reverse the effect.

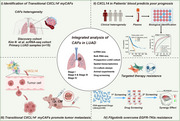

## 1 INTRODUCTION

Lung adenocarcinoma (LUAD) as the most common histological subtype of lung cancer induces more than one million deaths per years.^1^ Unfortunately, almost 70% LUAD patients present with advanced‐stage disease with lymphatic or haematogenous metastasis at the first diagnosis.^2^ Metastasis is a dynamic, multistep process influenced by the tumour microenvironment (TME), wherein normal cells undergo transformation into cancerous cells. These oncogenic cells exhibit uncontrolled proliferation, evade detection by the immune system, resist apoptosis, promote the formation of new blood vessels, develop invasive characteristics, survive circulation within the bloodstream and form malignant tumours in distant organs.^3^, ^4^ Understanding the key drivers of this process is essential for developing effective therapies. Cancer‐associated fibroblasts (CAFs) are prevailing stromal cells in the TME of LUAD. Recent research has demonstrated that CAFs exhibit substantial molecular heterogeneity, with various subsets fulfilling distinct roles in tumour development.^5^, ^6^ For instance, in pancreatic and breast cancers, there are notable examples of different CAF subsets. Myofibroblastic CAFs (myCAFs), characterised by high levels of αSMA and associated with extracellular matrix (ECM) signatures, are typically found adjacent to cancer cells. In contrast, inflammatory CAFs (iCAFs) show low levels of αSMA but express elevated amounts of cytokines and chemokines, and they are generally located further away from the tumour cells. Additionally, antigen‐presenting CAFs (apCAFs) are defined by their high expression of major histocompatibility complex (MHC) class II and CD74, playing a significant role in shaping tumour immunity.^7^, ^8^, ^9^

Mechanistically, CAFs can release a variety of soluble proteins, including growth factors, cytokines and components of the ECM. These secreted molecules contribute to promoting tumour cell growth, enhancing tumour aggressiveness and facilitating the formation of pre‐metastatic niches.^10^ Moreover, CAFs have also been shown to promote resistance in lung cancer cells to clinical standard therapies, such as chemotherapy,^10^, ^11^ epidermal growth factor receptor‐tyrosine kinase inhibitors (EGFR‐TKIs)^12^, ^13^, ^14^, ^15^ or immunotherapy.^16^, ^17^, ^18^ However, there is currently a lack of systematic investigation into how these CAF subsets drive resistance to these therapies. Advancements in single‐cell RNA sequencing (scRNA‐seq) and spatial transcriptomics (ST) have enabled researchers to investigate transcriptional programs and cellular interactions within distinct TMEs. These technologies offer valuable tools for examining the heterogeneity of CAFs over time and in various spatial contexts.

In this study, we utilised multiple scRNA‐seq datasets and ST profiles to comprehensively decipher the specific CAF subsets in different clinical stages of LUAD. Notably, we identified a transitional myCAFs subset characterised by high expression of CXCL14 that predominantly existed in the advanced LUAD tissue and promotes tumour invasion and metastasis through enhancing epithelial–mesenchymal transition (EMT) and angiogenesis. Besides, we confirmed that CXCL14 in peripheral blood plasma as the biomarkers demonstrated excellent diagnostic efficacy for tumour progression. Strikingly, among the clinical standard therapies, transitional CXCL14^+^ myCAFs specifically conferred resistance to EGFR‐TKIs therapy, rather than chemotherapy or immunotherapy. Furthermore, screening a library of US Food and Drug Administration (FDA)‐approved small molecular compounds revealed that filgotinib synergises with EGFR‐TKIs to selectively inhibit LUAD models that exhibit resistance enabled by transitional CXCL14^+^ myCAFs.

## 2 RESULTS

### 2.1 Identification of a transitional CXCL14^+^ myCAFs subset associated with advanced‐stage LUAD

**FIGURE 1:**
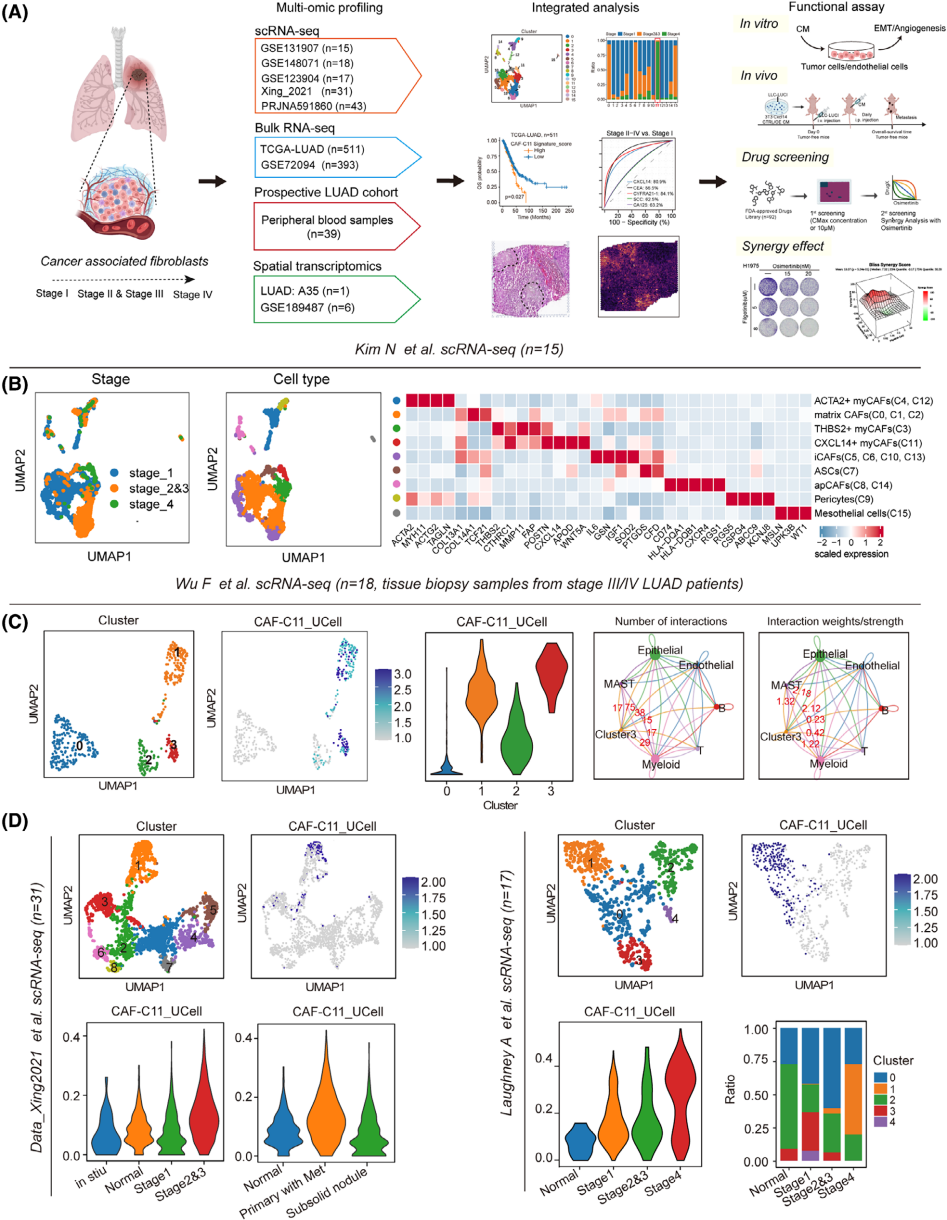
Single‐cell analysis uncovers distinct cancer‐associated fibroblast (CAF) cell populations within human lung adenocarcinoma (LUAD). (A) Study design. We integrated five single‐cell RNA sequencing (scRNA‐seq) including Kim et al. cohort (*n* = 15), Xing et al. cohort (*n* = 31), Laughney et al. cohort (*n* = 17), Wu et al. cohort (*n* = 18) and Maynard et al. cohort (*n* = 43), bulk RNA‐seq data obtained from 511 LUAD samples, which include information on overall survival (OS), sourced from The Cancer Genome Atlas (TCGA) database; 393 LUAD samples from GSE72094, peripheral blood samples (*n* = 39) measured by enzyme‐linked immunosorbent assay (ELISA) and spatial transcriptomics data from our centre (*n* = 1) and GSE189487 (*n* = 6), we focused on CXCL14^+^ myCAFs associated with tumour progression, treatment response and prognosis. Following this, in vitro and in vivo experimental assays were conducted. Through a wide array of supporting evidence, we elucidated the intricate molecular interactions and physiological roles associated with the identified myCAFs. (B) (1) Uniform Manifold Approximation and Projection (UMAP) plots of fibroblast cells show sample stages. (2) UMAP plots categorise fibroblast cells into clusters. (3) Violin plot illustrates marker gene expression among various CAF subclusters. (C) (1) UMAP plots represent fibroblast cells, with clusters colour‐coded as instructed. (2) Featureplot colour‐coded for CAF‐C11 signature scores. (3) Violin plots display normalised expression levels of signature scores for CAF‐C11, across each fibroblast subcluster. (4) Numbers and weights of significant ligand‐receptor pairs between Cluster3 and other cell types. (D) For Xing et al. cohort: (1) UMAP plots illustrate fibroblast cells with colour‐coded clusters. (2) Featureplot colour‐coded for CAF‐C11 signature scores. (3, 4) Violin plot showing expression of CAF‐C11 signature scores for specific subgroups. For Laughney et al. cohort: (1) UMAP plots illustrate fibroblast cells with colour‐coded clusters. (2) Featureplot colour‐coded for CAF‐C11 signature scores. (3) Violin plot showing expression of CAF‐C11 signature scores for stage subgroups. (4) Cellular composition of all fibroblast clusters in different stages.

To identify the specific CAF subsets in different clinical stages of LUAD, we obtained four independent scRNA‐seq datasets of LUAD, including the study by Kim et al.^19^ (*n* = 15), the study by Xing et al.^20^ (*n* = 31), the study by Laughney et al.^21^ (*n* = 17) and the study by Wu et al.^22^ (*n* = 18; Figure 1A). Following this, we used the Kim et al.’s dataset as a discovery cohort and the other datasets as the validating datasets. To accurately identify characteristics of CAFs in primary tumour lesions, we firstly included primary LUAD samples (*n* = 15) from the Kim et al.’s dataset and excluded those from metastatic sites. We conducted dimensionality reduction and clustering and identified 19 clusters. The identified clusters were annotated into T cells, NK cells, B cells, myeloid cells, mast cells, fibroblasts, endothelial cells and epithelial cells according to the expression of specific marker genes and the signature genes within each cluster with established cell population markers found in the literature (Figure S1C and Table S1).

Next, we specifically investigated cellular heterogeneity within the CAF subpopulations (*n* = 1813). Using the ‘Clustree’ algorithm^23^ to determine the optimal cluster number, we identified 16 subclusters of CAFs through unsupervised graph‐based clustering, visualised via Uniform Manifold Approximation and Projection (UMAP) algorithm (Figure S1D). Based on the gene signature derived from these CAFs subsets (Table S2), our analysis revealed that four clusters (0, 1, 3 and 11) consistently exhibited high expression levels in LUAD tumours when compared to normal lung tissue in several LUAD cohorts.^24^, ^25^, ^26^, ^27^, ^28^, ^29^, ^30^ In contrast, the other 12 clusters showed substantial downregulation, despite some variations (Figure S1E–K). Subsequently, we identified nine major functional subpopulations including ACTA2^+^ myCAFs (C4, C12), matrix CAFs (C0, C1, C2), THBS2^+^ myCAFs (C3), CXCL14^+^ myCAFs (C11), iCAFs (C5, C6, C10, C13), adipose stromal cells (ASCs; C7), apCAFs (C8, C14), pericytes (C9) and mesothelial cells (C15) based on marker genes (Figure 1B). We investigated which CAFs subclusters in LUAD were most associated with the tumour pathological stage, a commonly used surrogate marker for tumour progression. Intriguingly, our previous study has identified THBS2^+^ myCAFs (C3) as a key orchestrator promoting aggressiveness in early‐stage LUAD, which is mostly manifested in stage I–III LUAD patients. Of note, we noticed that CXCL14^+^ myCAFs (CAF‐C11), another subset of myCAFs, mainly existed in the advanced‐stage LUAD tumours (Figure 1B). Hallmarks gene set variation analysis (GSVA) demonstrated significant enrichment of EMT and angiogenesis signatures for the CAF‐C11 subset (Figure S1L). Furthermore, the association between the enrichment levels of these two pathways (EMT and angiogenesis; Table S3) and pathological stage was also evident in The Cancer Genome Atlas (TCGA) LUAD database^24^ (Figure S1M). Notably, the differentially expressed genes (DEGs) and functional enrichment revealed that C11 (e.g., *CXCL14*, *TWIST2*, *TSPAN8* and *APOD*) harbouring the activation of cytokine‐cytokine receptor interaction and chemokine signalling pathway, and C3 (e.g., *FN1*, *FXYD5* and *GPC6*) harbouring the activation of pathogenic *Escherichia coli* infection and ECM receptor interaction (Figure S1N). Trajectory inference analysis using Monocle2 further indicated a transition from C11 to C3, during which C11‐associated genes (e.g., *CXCL14*, *TWIST2*, *WNT5A* and *APOD*) exhibited a progressive decline in expression, while THBS2‐associated genes (e.g., *THBS2*, *COL11A1*, *FAP*, *POSTN* and *MMP11*) gradually increased (Figure S1O). Besides, Zhu et al.^31^ also demonstrated such co‐expression occurred at the intermediate stage of the differentiation of the APOD^+^ progenitor cells into the COL11A1^+^ THBS2^+^ CAFs. These findings suggest that CXCL14⁺ myCAFs within C11 represent a transitional state in the early‐stage differentiation towards invasiveness‐associated THBS2^+^ POSTN^+^ COL11A1^+^ myCAFs.

To further demonstrate the presence of molecular and functional features resembling transitional CXCL14^+^ myCAFs (CAF‐C11) in the advanced‐stage LUAD, we employed 18 stage 3/4 LUAD samples from the study by Wu et al.^22^ and performed clustering of 488 CAFs using the Clustree algorithm, which revealed the presence of 4 CAF subpopulations (Figure 1C). We then utilised the top 20 markers specific to transitional CXCL14^+^ myCAFs (CAF‐C11) compared to other CAFs subtypes as the gene signature (Table S2), leading to identifying that new Cluster 3 have the highest transitional CXCL14^+^ myCAFs score (Figure 1C).

In addition, the results from CellChat indicated that Cluster 3 predominantly interacted with epithelial cells and endothelial cells, further supporting its pivotal role in interaction with cancer cells and angiogenesis (endothelial cells; Figure 1C). Besides, we analysed two more single‐cell datasets spanning different stages of tumour progression. Clustering identified nine CAF clusters and five CAF clusters, respectively (Figure 1D) and featureplot obtained that new Cluster1 in these two datasets had the highest signature score of transitional CXCL14^+^ myCAFs (Figure 1D). Also, new Cluster 1 was more abundant in samples from patients in the late stages (Figure 1D). These data provided evidence for the presence of transitional CXCL14^+^ myCAFs in advanced‐stage LUAD.

### 2.2 Transitional CXCL14^+^ myCAFs are associated with unfavourable prognosis and tumour metastasis in LUAD patients

**FIGURE 2:**
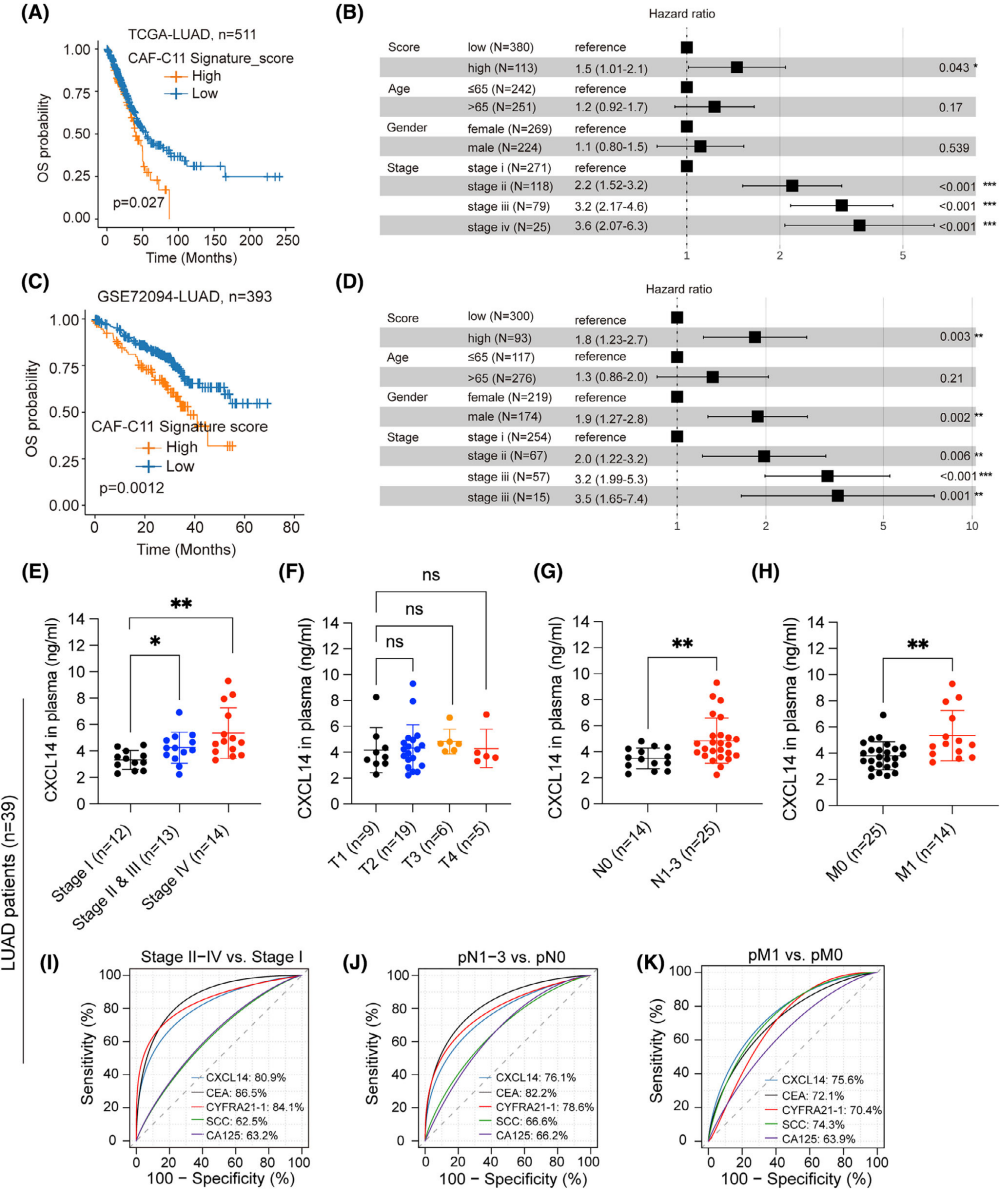
Cancer‐associated fibroblast (CAF)‐C11 (transitional CXCL14^+^ myofibroblastic CAFs [myCAFs]) correlates with poor patient prognosis. (A–D) Kaplan–Meier survival curves for lung adenocarcinoma (LUAD) patients were generated based on CAF‐C11 signatures, dividing them into groups as indicated, across two LUAD cohorts, including TCGA‐LUAD (*n* = 511) and GSE72094 (A, C). Multivariate Cox analysis of clinicopathologic and signature score characteristics showed shown in B and D. *p*‐values, hazard ratios and 95% confidence intervals represented. (E–H) Enzyme‐linked immunosorbent assay (ELISA) was used to detect the expression levels of CXCL14 in the plasma of patients at different pathological stages. An unpaired *t*‐test was used for statistical analysis. **p < .01 and *p < .05. (I–K) Comparison of the difference between the receiver operating characteristic (ROC) curves of five predictive models (CXCL14, CEA, CTFRA21‐1, SCC and CA125).

We then investigated the clinical significance of transitional CXCL14^+^ myCAFs in multiple LUAD cohorts. The survival analysis based on the gene signature of transitional CXCL14^+^ myCAFs (Table S2) showed that a higher score was associated with shorter OS in TCGA‐LUAD^24^ (*n* = 511) and GSE72094^32^ (*n* = 393; Figure 2A,C). Multivariable Cox regression analyses demonstrated the independent prognostic significance of transitional CXCL14^+^ myCAFs infiltration in OS of patients with LUAD (Figure 2B,D).

Risk stratification is crucial for identifying high‐risk patients in the early clinical stages. Based on the correlation of transitional CXCL14^+^ myCAFs with tumour staging, we prospectively collected plasma samples from stage I (*n* = 12), stage II and III (*n* = 13) and stage IV (*n* = 14) LUAD patients (patients information shown in Table S5) to measure the cytokine CXCL14. The plasma expression levels of CXCL14 showed significant correlation with the tumour's pathological stages, the degree of lymph node involvement (N staging) and the presence of metastasis (M staging; Figure 2E–H). Notably, CXCL14 demonstrated superior predictive performance compared to commonly used clinical tumour markers by ROC curves (Figure 2I–K), highlighting its substantial predictive importance. Collectively, our analysis demonstrates that transitional CXCL14^+^ myCAFs facilities invasion and angiogenesis, which synergistically contribute to tumour progression.

### 2.3 Spatial transcriptomics reveals an interaction between transitional CXCL14^+^ myCAFs and tumour EMT and angiogenesis in advanced‐stage LUAD

**FIGURE 3:**
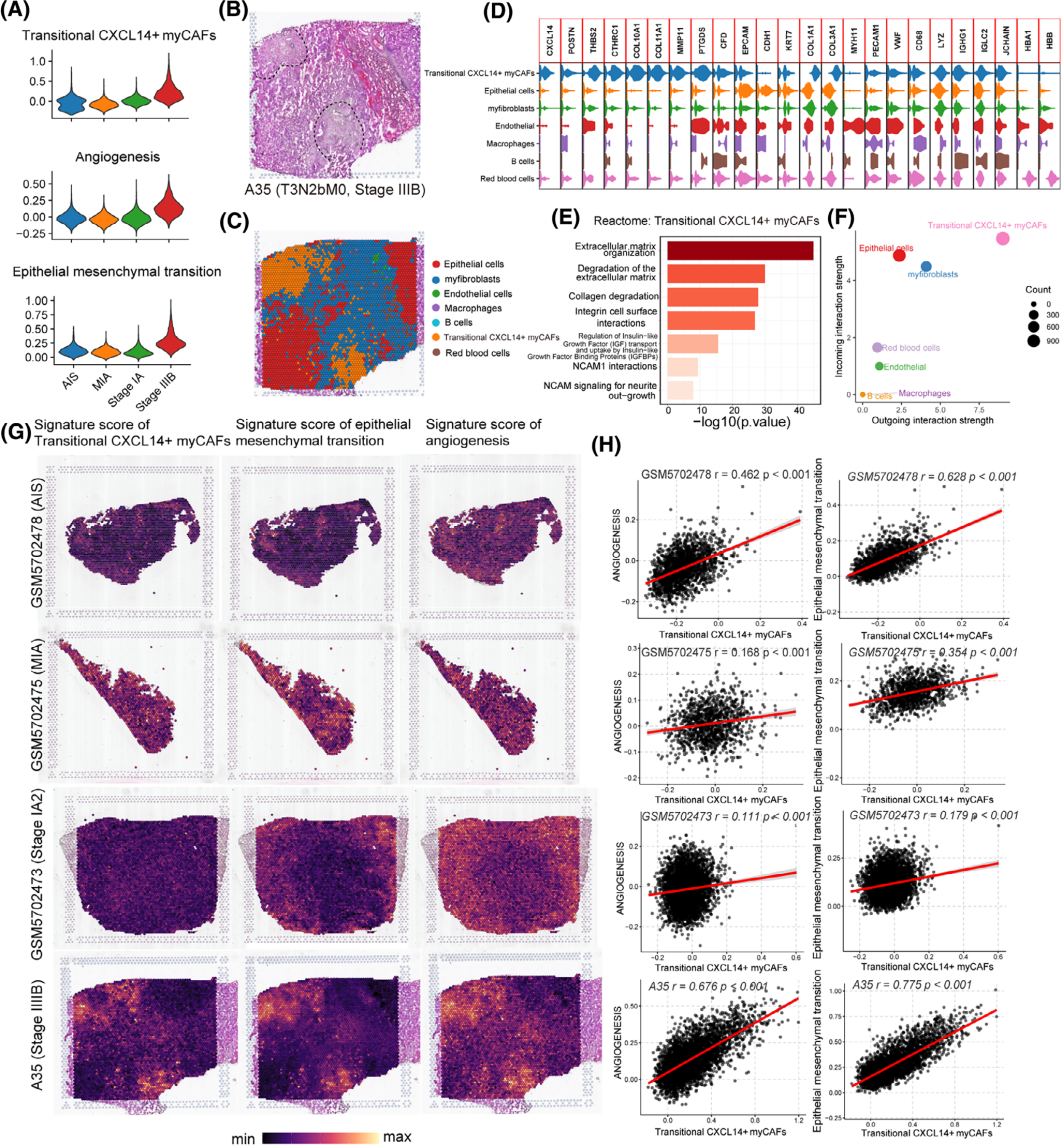
Spatial transcriptomics (ST) confirms transitional CXCL14^+^ myofibroblastic CAFs (myCAFs) induce epithelial–mesenchymal transition (EMT) and angiogenesis in advanced‐stage lung adenocarcinoma (LUAD). (A) Violin plots display the gene signatures of transitional CXCL14^+^ myCAFs, EMT and angiogenesis in different stages of LUAD. (B) Haematoxylin and eosin (H&E) staining of tumour tissue of A35 patient (stage IIIB). (C) Unbiased clustering of ST spots and definition of cell types in A35 patient. (D) Violin plots display the expression level of marker genes in each cell type. (E) Bar plot showing the pathway enrichment in transitional CXCL14^+^ myCAFs. (F) Activities of cells acting as sender cells (x axis) or receiver cells (y axis) in A35 patient based on CellChat. (G) Spatial feature plots of signature score of transitional CXCL14^+^ myCAFs, EMT and angiogenesis in GSM5702478 (AIS), GSM5702475 (MIA), GSM5702473 (stage IA2) and A35 (stage IIIB). (H) The Pearson correlation of signature score of transitional CXCL14^+^ myCAFs with EMT and angiogenesis in GSM5702478 (AIS), GSM5702475 (MIA), GSM5702473 (stage IA2) and A35 (stage IIIB).

To investigate whether transitional CXCL14^+^ myCAFs interact with EMT tumour cells and endothelial cells on the spatial level, we performed ST in an advanced‐stage LUAD section (A35, stage IIIB, pT3N2bM0; patients information shown in Table S6) from our centre. In addition, we integrated this section with six ST sections of early‐stage LUAD patients (including two cases of adenocarcinoma in situ [AIS], two cases of minimally invasive adenocarcinoma [MIA], and two cases of invasive adenocarcinoma [IAC]) from the GSE189487 dataset.^33^ After batch correction and the UMAP clustering, we found that the scores of transitional CXCL14^+^ myCAFs, EMT and angiogenesis which were highly enriched in advanced‐stage LUAD section compared with early‐stage LUAD (Figure 3A). Then, the A35 section was identified into seven cell types including epithelial cells, transitional CXCL14^+^ myCAFs, myofibroblasts, endothelial cells, macrophages, B cells and red blood cells (Figure 3B). The subcluster of transitional CXCL14^+^ myCAFs specially expressed typical marker genes of myCAFs (*POSTN*, *CTHRC1* and *COL10A1*) and CXCL14 (Figure 3C,D). Reactome analysis revealed that pathways of ECM organisation, collagen degradation, NCAM1 interactions and so forth activated in the transitional CXCL14^+^ myCAFs (Figure 3E). In patient A35, the CellChat analysis confirmed robust cell–cell interactions by demonstrating that transitional CXCL14^+^ myCAFs exhibited the highest levels of activity as both receiver (receptor) and sender (ligand; Figure 3F). The score plots in each cluster with gene signatures from scRNA‐seq data revealed that transitional CXCL14^+^ myCAFs co‐located with angiogenesis and EMT in the same cluster (Figure 3G). In addition, we found a more significantly positive correlation between the signature score of transitional CXCL14^+^ myCAFs and the pathways of angiogenesis and EMT in advanced‐stage LUAD section (Figure 3H). The direct contact and close interaction between transitional CXCL14^+^ myCAFs and promoting metastasis factors, such as EMT tumour cells and angiogenesis, were revealed by the spatial data.

### 2.4 Elevated Cxcl14 in fibroblasts induced proliferation and metastasis of LUAD

**FIGURE 4:**
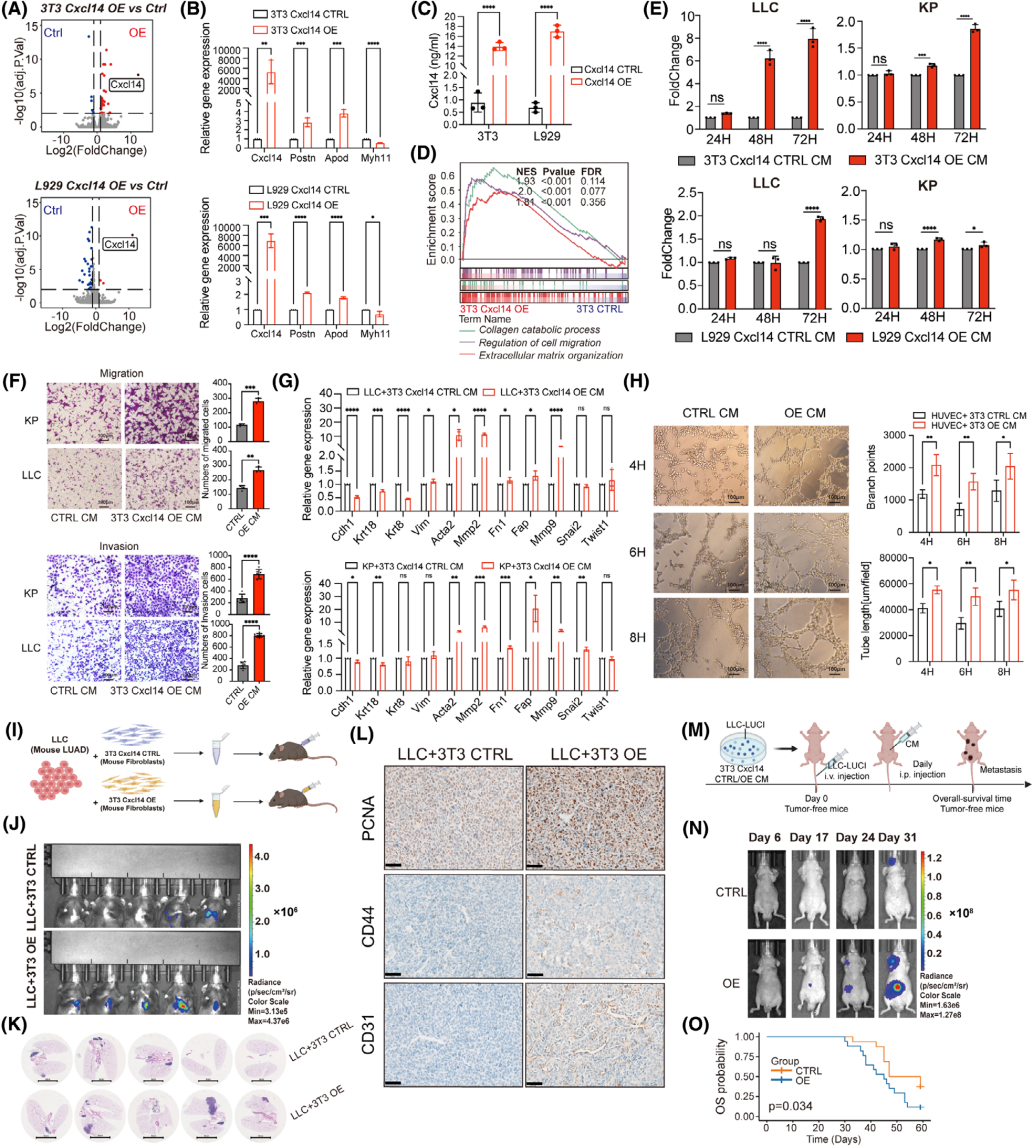
Cancer‐associated fibroblast (CAF)‐C11 (transitional CXCL14^+^ myofibroblastic CAFs [myCAFs]) emerges as a key player in facilitating tumour metastasis by stimulating angiogenesis and promoting epithelial–mesenchymal transition (EMT). (A) Volcano plot illustrates the genes that are differentially expressed between 3T3/L929 control and Cxcl14‐OE cells. (B) Quantitative reverse‐transcriptase polymerase chain reaction (qRT‐PCR) was performed to assess the mRNA levels of *Cxcl14*, *Postn*, *Apod* and *Myh11* genes in 3T3/L929 control and Cxcl14‐OE cells. (C) Enzyme‐linked immunosorbent assay (ELISA) was used to detect the expression levels of Cxcl14 in the conditioned medium (CM) of 3T3/L929 control and Cxcl14‐OE cells. (D) Gene set enrichment analysis (GSEA) was conducted on RNA‐seq data obtained from 3T3 control and 3T3 Cxcl14‐OE cells, revealing notable enrichment in pathways related to collagen catabolic process, regulation of cell migration and extracellular matrix organisation. (E) The growth of Lewis lung carcinoma (LLC) and KP cells in CM collected from fibroblasts control or Cxcl14‐OE cells. Results are presented as a fold of control. (F) In vitro transwell assays were performed on LLC/KP cells exposed to CM obtained from 3T3/L929 control or Cxcl14‐OE cells. (G) qRT‐PCR analysis was conducted on LLC and KP cells following co‐culture with CM from either 3T3 control or Cxcl14‐OE cells. The analysis included an assessment of transcript levels for genes encoding epithelial markers (*Cdh1*, *Krt18* and *Krt8*), mesenchymal markers (*Vim*, *Acta2*, *Mmp2*, *Fn1*, *Fap* and *Mmp9*) and EMT transcription factors (*Snail2* and *Twist1*; *n* = 3). (H) Primary umbilical vein endothelial cells (HUVECs) tube formation over time after co‐cultured with CM from 3T3 control and Cxcl14‐OE cells. Representative images and quantified data using indicators such as branch points and tube length were presented. (I) C57BL/6 mice received injections of LLC‐luc cells alongside Cxcl14‐OE or control 3T3 cells for subcutaneous and orthotopic xenograft experiments. (J) In vivo bioluminescence images capturing the orthotopic xenografts of LLC‐luc cells (*n* = 5). (K) Visuals showcased representative haematoxylin and eosin (H&E)‐stained lung tissues treated with either the control or overexpression groups. (L) Representative immunohistochemistry (IHC) staining of PCNA, CD44 and CD31. Bar, 50 µm. (M) LLC‐luc cells were primed for 72 h in CM obtained from either 3T3 control or Cxcl14‐OE cells prior to injection into the tail vein of BALB/c nude mice (*n* = 16–17). Daily intraperitoneal injections of 300ul CM from 3T3 control and Cxcl14‐OE cells were administered to mice for 6 weeks. (N) Two groups of tumour metastasis were monitored by luciferase signal intensities on the 6th, 17th, 24th and 31st days. (O) Mice were assessed for survival (*n* = 16–17). The data are presented as the means ± SD (B, C, E–H). *p*‐values were derived from unpaired two‐sided Student's *t*‐test (B, C, F, G) and two‐way analysis of variance (ANOVA) test (E, H). ****p < .0001; ***p < .001; **p < .01; and *p < .05.

Next, we conducted functional assays to explore the interplay between transitional CXCL14^+^ myCAFs and the pathways of angiogenesis and EMT. Overexpression (OE) of *Cxcl14* in two mouse fibroblast cells (NIH/3T3 and NCTC clone 929, abbreviated as 3T3 and L929 respectively thereafter) confirmed by RNA‐sequencing and enzyme‐linked immunosorbent assay (ELISA) data, quantitative reverse‐transcriptase polymerase chain reaction (qRT‐PCR) data revealed that *Cxcl14‐*OE was accompanied by increased *Postn*, *Apod* and decreased *Myh11* expression (Figure 4A–C). In detail, 3T3 and L929 Cxcl14‐overexpressed (Cxcl14‐OE) cells grew faster than control cells (Figure S2A). Functionally, the Cxcl14‐OE in lung fibroblasts notably enhanced their migration capacities (Figure S2B). Based on the RNA‐sequencing data, we observed that Cxcl14‐OE 3T3 cells were characterised by enrichment for collagen catabolic process, ECM organisation and regulation of cell migration (Figure 4D). In the co‐culture assay, conditioned medium (CM) derived from Cxcl14‐OE cells of 3T3 or L929 exhibited greater potency in promoting growth (Figures 4E and S2C) and migration (Figure 4F and S2D) of mouse LUAD cells (Lewis lung carcinoma [LLC] and KP cells (*KRAS^G12D^/TP53^−/−^*)) compared to that from control cells. Besides, flow cytometry analysis revealed that Cxcl14‐OE cells promoted the higher proportions of tumour cells in the S phase (Figure S2E) while could not significantly affect the apoptosis process (Figure S2F). In conjunction with this, significant enhancements in EMT markers were observed in LLC and KP cells co‐cultured with Cxcl14‐OE fibroblasts‐derived CM (Figure 4G). In addition, experimental evidence on angiogenesis demonstrates that human umbilical vein endothelial cells (HUVECs) co‐cultured with CM from 3T3 Cxcl14‐OE exhibit enhanced tube‐forming ability (Figure 4H).

Next, we performed the co‐injection of 3T3 Cxcl14‐OE cells with mouse LLC cells, either subcutaneously or orthotopically, into immune‐competent C57BL/6 mice led to a substantial enhancement in tumour growth compared to the control group (Figures 4I–K and S2G,H). The immunohistochemistry (IHC) staining showed significant enhancements of PCNA+ cells, CD44+ cells and CD31+ cells in the group of 3T3 Cxcl14‐OE cells with mouse LLC cells in the orthotopical model (Figure 4L). Furthermore, using a mouse metastasis model induced by tail vein injection of cancer cells described before (Figure 4M), CM derived from Cxcl14‐OE fibroblasts promoted LUAD tumour metastasis (Figure 4N), with decreased survival (Figure 4O). In parallel, intravenous co‐injection of a mixture of cancer cells and fibroblasts failed to recapitulate this effect (Figure S2I). This discrepancy could be attributed to the disruption of interactions or communications between fibroblasts and cancer cells within the circulatory system immediately following co‐injection. Overall, these findings indicated that transitional CXCL14^+^ myCAFs could enhance the EMT in tumour cells and promote the angiogenesis of vascular endothelial cells.

### 2.5 Transitional CXCL14^+^ myCAFs are associated with EGFR‐TKIs resistance in LUAD patients

Chemotherapy, EGFR‐TKIs‐targeted therapy and immunotherapy are common systemic treatments for LUAD. To explore whether transitional CXCL14^+^ myCAFs are associated with clinical therapies, we analysed different datasets covering these three common therapies. Through the analysis of multiple chemotherapy and immunotherapy cohorts, we found that CAF‐11 signature (listed in Table S2) cannot predict the efficacy of these two treatment modalities (Figure S3A–F). Additionally, we analysed a scRNA‐seq dataset,^34^ spanning across three different EGFR‐TKIs treatment stages: prior to the commencement of systemic targeted therapy (TKI naïve [TN]), during the residual disease (RD) state and upon developing acquired drug resistance (progression disease [PD]). Of note, the RD state represents a residual lesion that cannot be eradicated after EGFR‐TKIs treatment, and the PD state indicates a de novo resistance of a tumour.

**FIGURE 5:**
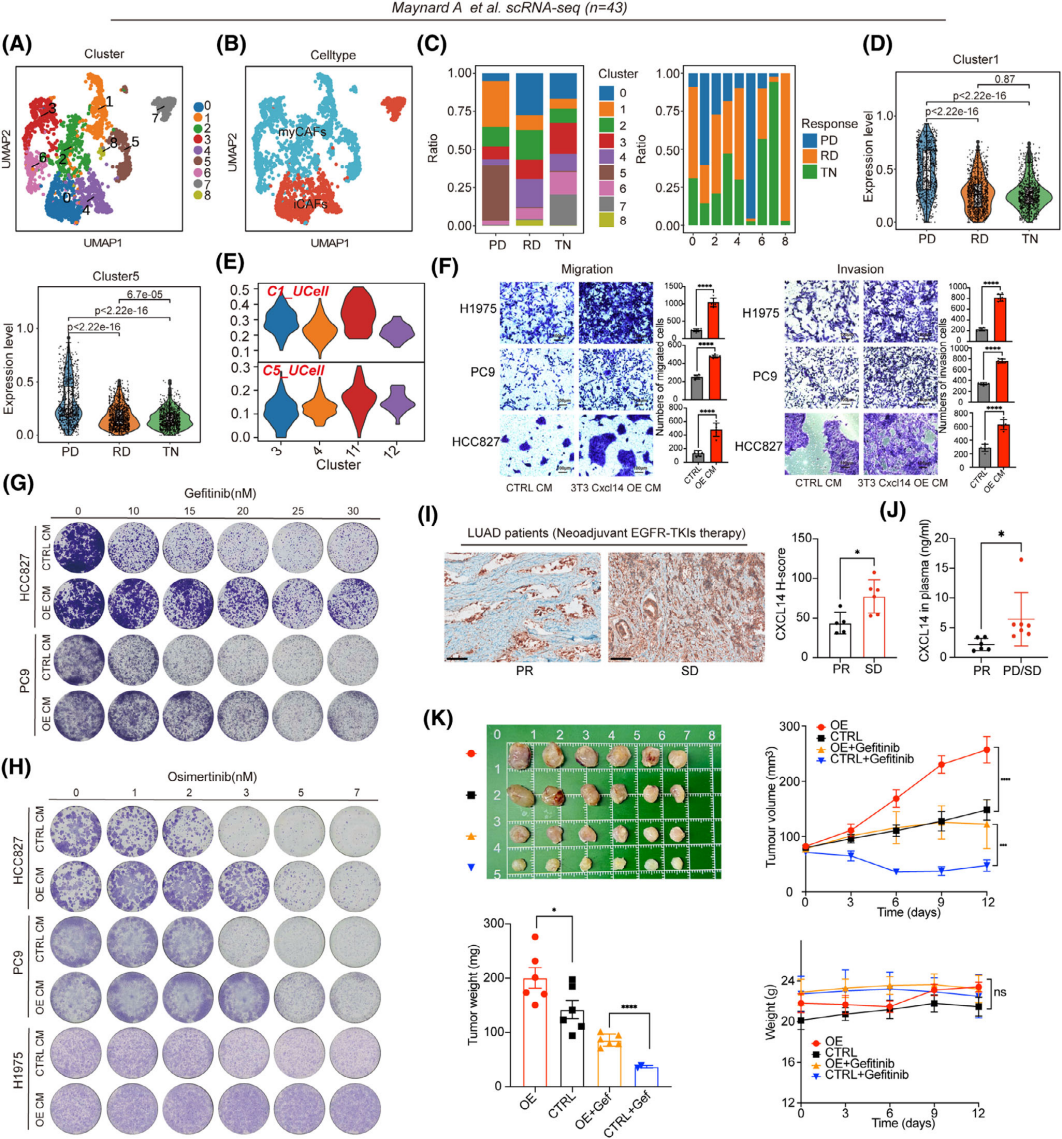
Cancer‐associated fibroblast (CAF)‐C11 (transitional CXCL14^+^ myofibroblastic CAFs [myCAFs]) is linked with resistance to targeted therapy. (A, B) Uniform Manifold Approximation and Projection (UMAP) plots depict fibroblast cells, with colour coding indicating clusters and cell subsets. (C) The distribution of CAF subsets from tissues at three different stages of epidermal growth factor receptor‐tyrosine kinase inhibitors (EGFR‐TKIs) treatment is presented: before initiating systemic targeted therapy (TKI naive [TN]), at the residual disease (RD) state, and upon acquired drug resistance (progression disease [PD]). TN, *n* = 14 samples; RD, *n* = 10 samples; PD, *n* = 19 samples. (D) Violin box plots of signature scores for Cluster 1 and 5 in PD, RD and TN patients. Unpaired two‐sided Student's *t*‐test was used. (E) Violin plots showing Cluster 1 and Cluster 5 signature scores by UCell across different myCAF subclusters. (F) In vitro transwell assays were performed on H1975, PC9 and HCC827 cells exposed to conditioned medium (CM) obtained from 3T3 control or Cxcl14‐OE cells. ****p < .0001 by unpaired Student's *t*‐test. (G, H) HCC827, PC9 and NCI‐H1975 cell lines underwent treatment with the indicated doses of EGFR‐TKI osimertinib and gefitinib, followed by colony formation survival assays. (I) Representative images of immunohistochemical staining for CXCL14 in neoadjuvant EGFR‐TKIs biopsies (left). Scale bars, 100 µm. CXCL14 *H*‐scores of neoadjuvant EGFR‐TKIs biopsies (*n* = 11) with different EGFR‐TKIs responses. Data expressed as mean ± SD, *p < .05 by unpaired Student's *t*‐test. (J) The expression level of CXCL14 in the plasma of patients with different EGFR‐TKIs responses was detected by enzyme‐linked immunosorbent assay (ELISA; *n* = 13). Mean ± SD, *p < .05 by unpaired Student's *t*‐test. (K) Representative tumour images, tumour volumes, tumour weight and mouse weight were shown (*n* = 6). The data are presented as the means ± SEM. ****p < .0001; ***p < .001 by two‐way analysis of variance (ANOVA) test (tumour volume and mouse weight), unpaired two‐sided Student's *t*‐test (tumour weight).

**FIGURE 6:**
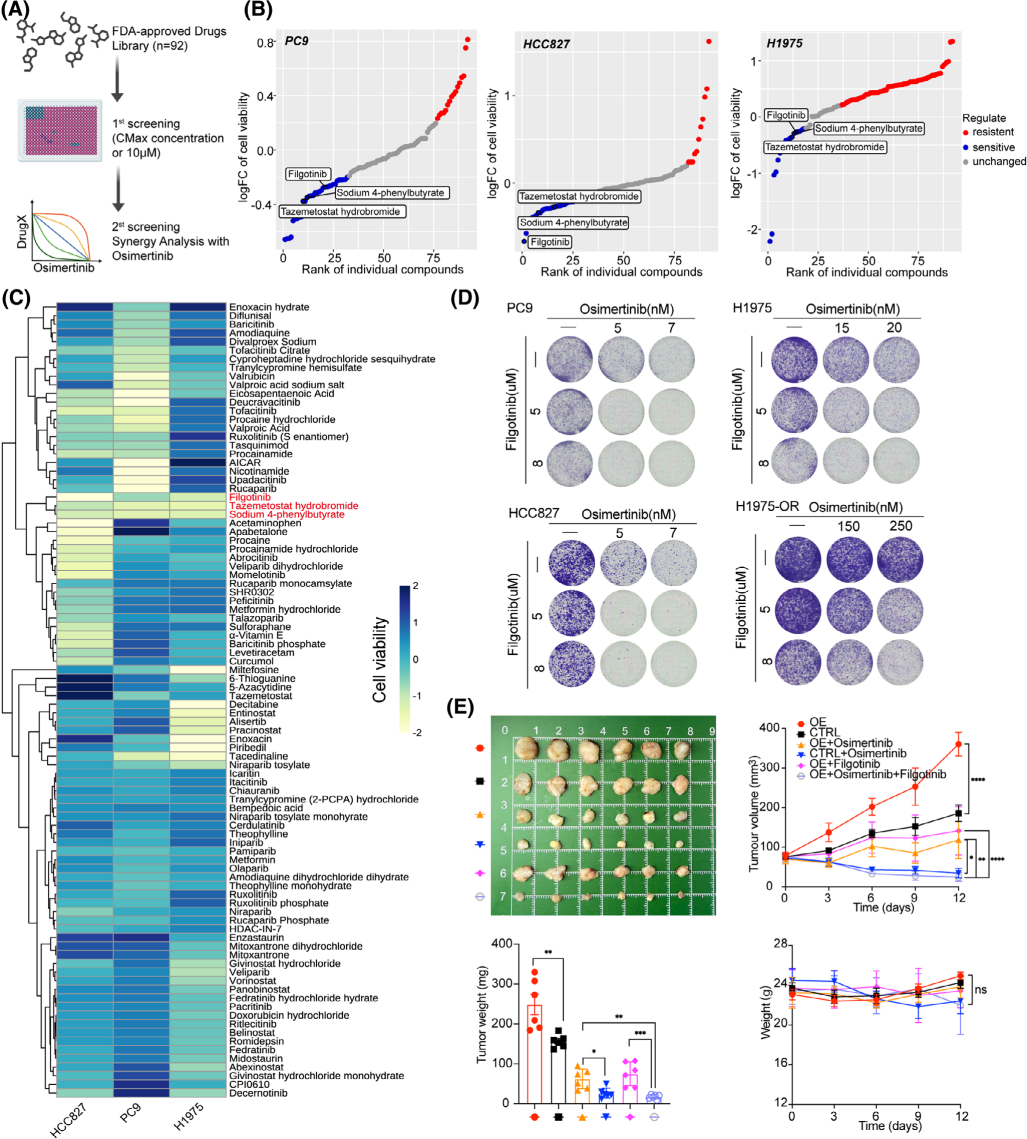
Osimertinib and filgotinib had synergistic effects. (A) The flowchart for drug screening is shown. (B) Hockey‐stick plots representing the compounds rank and logFC cell viability in PC9, HCC827 and H1975 cell lines. Representative compounds that are sensitive in the overexpression group compared to tumour cells cultured with control conditioned medium (CM) are shown in blue. (C) Heatmaps reflecting normalised score values of the cell viability of the overexpression group minus that of the control group in PC9, HCC827 and H1975 cell lines. (D) Representative colony formation assay in PC9, HCC827, H1975 and H1975‐OR cells after treated with osimertinib and filgotinib. (E) Representative tumour images, tumour volumes, tumour weight and mouse weight were shown (*n* = 6). The data are presented as the means ± SEM. ****p < .0001; ***p < .001 by two‐way analysis of variance (ANOVA) test (tumour volume and mouse weight), unpaired two‐sided Student's *t*‐test (tumour weight).

After clustering and annotation of the CAFs (*n* = 2493; Figure 5A,B), we observed that within this new dataset, Cluster 1 and Cluster 5 cells comprised a major fraction of all CAFs in the samples with PD compared to those with TN or RD (Figure 5C). Notably, based on the gene signatures, which comprised the top 20 markers derived from each CAF subcluster of this dataset (Table S7), we observed that CAFs from PD samples displayed significantly elevated scores of C1 and C5 (Figure 5D), suggesting that C1‐ and C5‐represented CAFs were associated with de novo therapy resistance to EGFR‐TKIs. Remarkably, through UCell, we observed that signature scores originating from C1 and C5 within this dataset were predominantly associated with the transitional CXCL14^+^ myCAFs (CAF‐C11; Figure 5E), indicating that this specific subset of myCAFs represents a resistance phenotype to EGFR‐TKIs. Indeed, culturing EGFR‐mutant LUAD cells (PC9, HCC827 and H1975 cells) using the CM derived from Cxcl14‐OE fibroblasts resulted in ECM and resistance to different generations of EGFR‐TKIs (including gefitinib, afatinib and osimertinib; Figures 5F–H and S3G,H). Further, we retrospectively selected tissue samples from LUAD patients receiving neoadjuvant EGFR‐TKIs therapy to assay the expression level of CXCL14. The IHC results revealed that stable disease (SD) patients had the higher *H*‐scores of CXCL14 than partial response (PR) patients (Figure 5I, patients information shown in Table S5). Besides, we collected plasma samples from LUAD patients receiving adjuvant EGFR‐TKIs therapy. Analogously, PD and SD patients had higher concentrations of CXCL14 in plasma than PR patients (Figure 5J, patients information shown in Table S5). Similarly, CM obtained from Cxcl14‐OE fibroblasts also facilitated the resistance of LLC (KRAS^G12C^) and KP (KRAS^G12D^) to KRAS^G12C^ (clinically approved MRTX849 and AMG510) and KRAS inhibitors (MRTX1133), respectively (Figure S3I). Besides. the co‐injection of 3T3 Cxcl14‐OE cells with human EGFR‐mutant LUAD cells exhibited tumour proliferation (Figures 5, 6 and S3J) and drug resistance of gefitinib and osimertinib in vivo (Figures 5K and 6E). Together, transitional CXCL14^+^ myCAFs could induce the drug resistance to EGFR‐TKIs.

### 2.6 Screening of FDA‐approved inhibitors based on the small‐molecule library

To find a small‐molecule inhibitor that has a synergistic effect with EGFR‐TKIs to overcome resistance, through a pharmacological screen encompassing FDA‐approved drugs (*n* = 92) in phase III clinical trials or clinical approval using the *C*_max_ concentration (if *C*_max_ exceeds 10 µM, use 10 µM as the initial screening concentration.), we identified that 3 clinically approved inhibitors (filgotinib, a JAK1 inhibitor; tazemetostat hydrobromide, a selective EZH2 inhibitor and sodium 4‐phenylbutyrate, an histone deacetylase [HDAC] inhibitor) that were sensitive to EGFR‐mutant LUAD cell lines co‐cultured with CM derived from 3T3‐Cxcl14 cells (Figure 6A–C), suggesting these inhibitors have the potential to overcome Cxcl14‐induced EGFR‐TKIs resistance.

Subsequently, we tested whether these three drugs had a synergistic effect with osimertinib to overcome resistance, we finally identified a JAK1 inhibitor, filgotinib could drastically overcome the CXCL14‐induced osimertinib‐resistant LUAD cells (Figures 6D and S4A). Filgotinib, an agent that selectively inhibits JAK1, is presently being studied in clinical trials for various ailments such as rheumatoid arthritis and inflammatory bowel disease.^35^ To date, it has demonstrated significant anti‐inflammatory effects, notably reducing levels of cytokines like IL‐6.^36^, ^37^, ^38^ Findings from research suggest that filgotinib may play a role in overcoming resistance to targeted treatment options, including therapies aimed at mitogen‐activated protein kinase (MEK), EGFR tyrosine kinase and anaplastic lymphoma kinase (ALK).^35^

To determine if filgotinib can effectively counteract secondary resistance when combined with osimertinib, we created one pair of parental and osimertinib‐resistant H1975 cell line. Parental cells showed an IC50 of 10.4 nM in response to osimertinib, while the resistant variants displayed IC50 values more than 500‐fold greater (Figure S4B). Proliferation of H1975‐OR cells was significantly increased compared to parental cells (Figure S4C). We evaluated the potential of JAK1 inhibition to sensitise a resistant cell line to osimertinib treatment by measuring drug synergy through Combenefit assays. Importantly, osimertinib and filgotinib had significant synergy in H1975‐OR cells (Figures 6D and S4D). We could not observe a striking synergy between osimertinib and tazemetostat hydrobromide (a selective EZH2 inhibitor) or sodium 4‐phenylbutyrate (an HDAC inhibitor; Figures 6D and S4D).

Subsequently, we explored the possibility of in vivo synergy between JAK1 inhibitors and osimertinib, utilising H1975 tumour cells integrated with either 3T3‐Cxcl14 vector or overexpression fibroblasts. Tumour‐bearing mice with tumour volumes of approximately 70 mm^3^ were randomly treated with vehicle, osimertinib (10 mg/kg, p.o.), filgotinib (5 mg/kg, p.o.) or the combination of osimertinib and filgotinib for 12 days (*n* = 6). While treatment with either drug alone led to only a slight reduction in tumour growth, the simultaneous use of osimertinib and filgotinib resulted in a significant reduction in tumour progression in vivo (Figure 6E). Additionally, Cxcl14 was found to promote tumour growth and contribute to resistance against osimertinib as described before (Figure 6E). Importantly, there were no notable changes in average body weight across the groups, suggesting the safety of the drug dosages used in the study (Figure 6E). Taken together, these results demonstrated that filgotinib combined with osimertinib could reduce the drug resistance induced by transitional CXCL14^+^ myCAFs.

## 3 DISCUSSION

CAFs have been extensively researched and are known to be crucial in tumour initiation, progression, metastasis and therapeutic resistance.^39^, ^40^, ^41^, ^42^ In this study, we integrated scRNA‐seq, bulk RNA sequencing datasets, ST and functional experiments, we proposed one CAFs subset (transitional CXCL14^+^ myCAFs) associated with metastasis, poor prognosis and EGFR‐TKIs resistance in LUAD patients, which might be instrumental for clinical patient stratification and optimisation of the therapy strategies. Assessment of the cellular makeup of CAF clusters using a discovery scRNA‐seq dataset with different clinical stages and genetic background led to the identification of transitional CXCL14^+^ myCAFs. To highlight biological heterogeneity, potential technical variation of scRNA‐seq analyses was minimised using multiple validated scRNA‐seq datasets and subsequent in vitro and in vitro experiments. Analysing a prospective cohort of blood samples from lung cancer patients across different stages, we uncovered that CXCL14 in plasma predicted the occurrence of metastasis and demonstrated similar diagnostic efficacy to typical tumour markers (e.g., CEA, Cyfra21‐1, SCC, etc.). Besides, we found that osimertinib combined with a JAK1 inhibitor, filgotinib could synergistically reverse the drug resistance induced by transitional CXCL14^+^ myCAFs using small‐molecule drug library screening. In summary, our approach provides additional insights into advanced‐stage LUAD complexity, potentially informing future therapeutic strategies.

Previous studies have suggested that CAFs can secrete a range of cytokines and metabolites, which promote tumour growth and EMT, ultimately contributing to cancer development.^43^ CXCL14, also referred to as BRAK (breast and kidney‐expressed chemokine), is a member of the CXC chemokine family.^44^ Recent studies have indicated that CXCL14 derived from CAFs has a cancer‐promoting effect, but epithelial‐derived CXCL14 mainly inhibits tumour progression.^45^ For instance, a previous study demonstrated that the fibroblast‐derived factor CXCL14 promoted the proliferation and migration of prostate cancer cells in vitro, as well as angiogenesis in vivo.^46^ Mechanically, this effect was attributed to the activation of actomyosin contractility and improved matrix remodelling capabilities and further promoted osteosarcoma lung metastasis in fibroblasts, initiated by the binding of CXCL14 to integrin α11β1.^47^ Additionally, CXCL14 facilitates EMT and subsequent cell migration in lung cancer by transactivating the ACKR2/PLC/PKC/c‐Src signalling pathway.^48^ In our research, we identified a subgroup known as transitional CXCL14^+^ myCAFs, which are exclusively present in advanced‐stage LUAD in multiple scRNA‐seq datasets. Based on the functional enrichment, clinical plasma samples and spatial features, we speculated their roles in tumour progression and metastasis through mechanisms such as angiogenesis and EMT. Specifically, CXCL14 is one of the key markers of a particular progenitor population present in naturally occurring ASCs, also found as fibro‐adipogenic progenitors (FAPs), which has recently been referred to as the ‘ASC/FAP population^49^’, and is characterised by a gene signature with prominent presence of gene APOD. Importantly, it undergoes differentiation into another particular population of CAFs, which gradually expresses POSTN and eventually expresses collagen COL11A1.^31^ We found a similar differentiation direction in LUAD, and pseudo‐sequential analysis showed that C11 (transitional CXCL14^+^ myCAFs) and C3 (THBS2^+^ myCAFs) had continuous differentiation characteristics including decreased level of *CXCL14*, *TWIST2*, *WNT5A* and *APOD and increased* level of *THBS2*, *POSTN*, *COL11A1*, *FAP* and *MMP11*. Supportably, we observed overexpressing *Cxcl14* in fibroblasts led to a higher expression level of *Postn*. Multiple studies emphasised that POSTN^+^ COL11A1^+^ INHBA^+^ THBS2^+^ myCAFs population participated in metastasis‐associated mechanism in lung cancer,^50^ pancreatic cancer^51^ and ovarian cancer.^52^ These observations indicated that CXCL14 may represent an earlier status in the differentiation process of POSTN^+^ COL11A1^+^ INHBA^+^ THBS2^+^ myCAFs, which provided us with a better therapeutic target. Finally, we demonstrated that elevate Cxcl14 in fibroblasts could mimic CXCL14^+^ myCAFs, which promoted the formation of EMT tumour cells, tube‐forming of vascular endothelial cells using functional assay. The advanced‐stage LUAD patients exhibited higher concentration of CXCL14, especially reflecting to the late N and M stage. Convincingly, intraperitoneal injection of CM from Cxcl14‐overexpressed fibroblasts promoted metastasis and poor prognosis eventually in nude mice.

Acquired resistance to EGFR‐TKIs can arise from mechanisms such as modifications in target genes, activation of alternative pathways and histological or phenotypic transformations.^53^ CAFs contribute to cancer cell resistance against EGFR‐TKIs through various processes, including ECM remodelling, the secretion of soluble molecules, exosomal vesicle delivery and metabolic interactions.^13^ For example, CAFs can over‐secret ANXA2, which triggers EMT in lung cancer cells; this pro‐EMT phenotype is dependent on the secretion of hepatocyte growth factor (HGF) and insulin‐like growth factor 1 (IGF1) by CAFs, thus facilitating the cancer cells' evasion of EGFR targeting.^54^ Using the scRNA‐seq dataset and similarity measurement of transcriptional features, transitional CXCL14^+^ myCAFs also exhibited enrichment in the PD patients. By demonstrating potential contribution to targeted therapy resistance, we proposed that the combined regimen of JAK1 inhibitor is a promising approach to overcome EGFR‐TKIs resistance using small molecular drug screening. The JAK‐STAT signalling pathway plays a significant role in the development of different cancers, such as non–small‐cell lung cancer (NSCLC), and impacts how effective EGFR‐TKIs are, as well as the mechanisms by which resistance to these therapies arises.^55^, ^56^, ^57^, ^58^, ^59^, ^60^ A clinical study investigated the combination of EGFR‐TKIs, such as osimertinib, with agents designed to block JAK activation. For patients possessing the T790 M mutation, the approach involved administering osimertinib in conjunction with JAK 1 inhibitors to interfere with the JAK/STAT signalling pathway within the framework of a second‐line therapeutic strategy. Additionally, a study conducted by Kim and colleagues revealed that the JAK1 inhibitor CJ14939 successfully reversed resistance to erlotinib, significantly enhancing the drug's ability to induce cell death in NSCLC cells that had developed resistance.^61^

However, the interferon signalling (JAK‐STAT) pathway is context dependent and has negative as well as positive roles in tumour control. In our study, we found that combining a JAK1 inhibitor with EGFR‐TKIs treatment enhances tumour control. This may be attributed to the compensatory activation of JAK1 signalling following EGFR‐TKIs treatment,^61^, ^62^ which likely arises as an adaptive response to EGFR signalling blockade and TME remodelling. It is unclear how JAK1 inhibitors affect the TME, especially the function of immune cells. Zak J et al. used high‐throughput screening to find that JAK inhibitors can reverse myeloid cells from immunosuppression to immunostimulation, thereby effectively rescuing exhausted T cell function, and performed well in a Phase I clinical study for patients with refractory/relapsed Hodgkin lymphoma treated with immunotherapy.^63^ Also, Mathew et al. found that JAK1 inhibitors improved T cell differentiation and function by blocking persistent type I interferon (IFN‐I) signals, and in a Phase II clinical study, the therapeutic effect of combined PD‐1 inhibitors for first‐line treatment of advanced NSCLC was two to three times that of existing first‐line immune treatments.^64^ The potential combination of JAK1 and PD1 inhibitors provides a potential therapeutic target for EGFR resistance. Clinically, persistent EGFR‐TKIs treatment could induce drug resistance and immune escape via activation of PD‐1 signalling. This characteristic of EGFR‐TKIs resistance exposed therapeutic vulnerability to immune‐related therapy. Multiple clinical trials including ALTER‐L308 (benmelstobart plus anlotinib),^65^ NCT02574078/CheckMate 370 (erlotinib and nivolumab)^66^ and NCT02039674/KEYNOTE‐021 (erlotinib /gefitinib and pembrolizumab),^67^ indicated that therapeutic potential of sequential immunotherapy after EGFR‐TKIs resistance.

In summary, our study of CAFs reveals their diverse roles in tumour progression, metastasis and targeted therapy, underscoring the importance of understanding CAF heterogeneity in stratifying LUAD patients. These findings offer valuable insights that could guide the development of more precise and effective cancer therapies in the future. However, further research and clinical validation are necessary to fully harness the therapeutic potential of targeting CAFs in LUAD treatment.

### 3.1 Data and code availability

This manuscript did not involve the creation of any new algorithms. One sample of self‐tested raw ST sequencing data and all code crafted for analysis can be obtained from the authors if requested.

### 3.2 Study declaration

The application of lung cancer tissue was sanctioned by the Institutional Review Board of the Shanghai Lung Tumor Clinical Medical Center, Shanghai Chest Hospital (Approval No.: KS(Y)23072), conforming to all pertinent ethical norms, such as the Helsinki Declaration as revised in 2013. Ahead of their participation in the research, comprehensive consent was secured from all the subjects involved. All procedures involving animals were executed in accordance with the Guide for the Care and Use of Laboratory Animals as published by the National Institutes of Health and received the approval from the Institutional Animal Care and Use Committee of Shanghai Jiao Tong University.

## 4 MATERIALS AND METHODS

### 4.1 Human sample acquisition

The clinical characteristics of the collected tumour samples used in ST sequencing are detailed in Table S6. The clinical information of the patients with plasma samples used for detecting CXCL14 is shown in Table S5.

### 4.2 Antibodies, primers and compounds

Table S9 contains detailed information on the antibodies, primers and compounds utilised in this study.

### 4.3 Cell lines and cell culture

The mouse LUAD cell line LLC (Lewis LLC cells) and LLC expressing firefly luciferase (LLC‐luc), as well as the human LUAD cell line HCC827, NCI‐H1975 and PC9, were preserved by our laboratory.^68^ The mouse fibroblast cell lines NIH 3T3 and NCTC clone 929 (abbreviated as 3T3 and L929, respectively) and HEK‐293T cells were provided by Prof. Hua Zhong at Shanghai Jiao Tong University. Primary umbilical vein endothelial cells (HUVECs) were provided by Wentao Fang at Shanghai Jiao Tong University. The GEMM‐derived mouse LUAD cell line KP (Kras−/− p53−/−) was provided by Yuezhen Deng at Shanghai Jiao Tong University. The cells were cultured in either Dulbecco's modified Eagle medium (DMEM) or RPMI‐1640 medium, both of which were supplemented with 10% foetal bovine serum (FBS), along with penicillin and streptomycin.

### 4.4 Construction of lentiviral vectors and viral transduction

Incorporation of Cxcl14 cDNAs into the Ubi‐MCS‐3FLAG‐CBh‐gcGFP‐IRES‐puromycin lentiviral vector was carried out. Lentiviral particles were then generated in HEK‐293T cells using two packaging plasmids (psPAX2 and pMD2G). Following this, 3T3 and L929 cells underwent transfection with Cxcl14 viruses, alongside respective control viruses, in the presence of polybrene. After transfection, cells expressing the desired constructs were selected using 2 mg/mL puromycin over a period of 1 week.

### 4.5 Fibroblast‐conditioned medium

Fibroblasts overexpressing Cxcl14, along with control fibroblasts, were cultured in 10 cm plates to a density of 7 × 10^5^ cells. After 72 h of incubation at 37°C, the CM was collected and sequentially centrifuged at speeds and durations of 300 × *g* for 10 min, 2000 × *g* for 10 min and finally 15 000 × *g* for 30 min. The CM was then sterile‐filtered using a. 44 µm Millipore Express PES Membrane Filter Unit and stored at −80°C.

### 4.6 Co‐culture assay

In six‐well plates, KP and LLC cells were cultured in a mixture of 1 mL DMEM medium with a 20% FBS concentration and 1 mL CM obtained from 3T3 or L929 control and Cxcl14‐OE cells. Every 24 h, the medium was replaced with a mixture comprising a 1:1 ratio of 2 mL 20% FBS medium and CM. After 48 h, cells were collected for qRT‐PCR to detect genes encoding EMT‐regulated markers.

### 4.7 In vitro growth assays

To analyse the impact of Cxcl14‐OE on fibroblast proliferation, 2 × 10^3^ Cxcl14‐OE and control fibroblasts were seeded in 96‐well plates. Cell viability was measured at 24 h, 48 h, 72 h and 96 h using Cell Titer Glo reagent. To conduct colony formation assays, fibroblast cells were seeded at a density of 5 × 10^3^ cells per well in a 12‐well plate, with medium changes performed daily. After 3 and 6 days, the cells were fixed using 4% paraformaldehyde (PFA) and subsequently stained with. 5% crystal violet.

To investigate the effect of fibroblast‐secreted Cxcl14 on tumour cell proliferation, 2 × 10^3^ tumour cells were seeded in 96‐well plates and cultured for 24 h before switching to 100 µL Cxcl14‐OE or control fibroblasts‐CM and 100 µL 20% FBS medium. Cell viability was measured at various time points using Cell Titer Glo reagent with a 200 µL volume, and luminescence was recorded using Bio Tek Synergy™ H1. The data were represented as Foldchange compared to the control group. For colony formation assays, 4 × 10^3^ tumour cells were seeded per well into a 12‐well plate. After 24 h, the medium was replaced with 2 mL per well (CM: 20% FBS DMEM = 1:1), and changed every 2 days. Every 2 days, the cells were stained with. 5% crystal violet after being fixed with 4% PFA.

### 4.8 In vitro migration and invasion assays

To study the cell migration of Cxcl14‐OE and control 3T3/L929 cells, a transwell migration assay was employed. Cells were subjected to an overnight fast in a medium containing only 1% FBS. Subsequently, 2 × 10^5^ 3T3/L929 control and Cxcl14‐OE (resuspended in 200 µL of FBS‐free medium) were seeded into transwell chambers with an 8.0‐µm pore‐size membrane, placed in a 24‐well plate filled with 30% FBS‐containing media (600 µL). For invasion assays, the upper chamber was coated with 70 µL of matrix gel diluted at a ratio of 1:3 and incubated at 37°C for 30 min. Discard the supernatant, and then 200 µL of medium containing 2 × 10^4^ fibroblasts were seeded into the upper chamber.

For the examination of tumour cell migration induced by Cxcl14 fibroblasts, 2 × 10^5^ tumour cells were reconstituted in the top compartment (filled with 200 µL of complete medium), while 600 µL of Cxcl14‐OE or control fibroblasts CM was added to the bottom chamber. For invasion assays, 200 µL of medium containing 2 × 10^4^ tumour cells were seeded into the upper chamber. After incubating for 6 h for migration assays and 48 h for invasion assays, cotton swabs were utilised to clean the interior of the insert. The cells were subsequently fixed in 4% PFA for 30 min, followed by three rinses with phosphate‐buffered saline (PBS) and staining with.5% crystal violet for 10 min. Images of cells were captured using a Zeiss microscope. Cellular migration was quantified by enumerating the nuclei of migrated cells.

### 4.9 RNA isolation, cDNA synthesis and qPCR

Cells were subjected to total RNA extraction using the UNIQ‐10 Column Trizol Total RNA Isolation Kit, followed by reverse transcription utilising the HiScript II Q RT SurperMix. The resulting cDNAs were utilised for qPCR on an ABI viia7 System, employing the ChamQ Universal SYBR qPCR Master Mix. Glyceraldehyde 3‐phosphate dehydrogenase (GAPDH) was used as the normalisation control for the obtained results. Table S8 provides a summary of the primers utilised for qRT‐PCR.

### 4.10 Enzyme‐linked immunosorbent assay

Fresh blood samples from patients were collected at Shanghai Chest Hospital, then centrifuged for 10 min at 1600 × *g*. The supernatant was collected as plasma, aliquoted and stored at −80°C to prevent repeated freeze–thaw cycles. Prior to testing, the samples were thawed and centrifuged again to eliminate any precipitate. Next, 100 µL of both standard and test plasma were added to each well and incubated at 37°C for 2 h. Following this, the liquid was discarded, and each well received 100 µL of a biotin‐labelled antibody working solution, which was then incubated at 37°C for another hour. After washing the plate three times, 100 µL of a horseradish peroxidase‐labelled avidin working solution was added to each well and incubated at 37°C for an additional hour. The plate was then washed five times before adding 90 µL of substrate to each well sequentially, followed by a 20‐min incubation at 37°C to allow for colour development. Finally, the reaction was halted by adding 50 µL of stop solution, and the optical density (OD value) of each well was measured at a wavelength of 450 nm.

### 4.11 Angiogenesis tube formation

In a 96‐well plate, 50 µL of Matrigel at a concentration of at least 10 mg/mL is added to each well and incubated at 37°C for 30 min to allow gelation. HUVEC cells pre‐cultured with CM for 48 h were resuspended in complete medium, and 100 µL of cell suspension containing 7 × 10^4^ cells was added to each well. The plate is then incubated for tube formation in the culture incubator, and images are captured at 2‐, 4‐ and 6‐h time points for observation. Quantitative analysis is performed using the Angiogenesis Analyzer plugin in Image J software.

### 4.12 In vitro cell viability assays

For cell viability assays, LLC and KP cells co‐cultured with CM obtained from 3T3 control or Cxcl14‐OE cells for 96 h were cultured overnight in a 96‐well plate at concentrations of 3 × 10^3^ and 1 × 10^3^ cells per well, respectively, to allow for adequate attachment.^69^, ^70^ After 24 h, 100 µL of medium derived from CM obtained from 3T3 control or Cxcl14‐OE cells containing various concentrations of inhibitors (MRTX849, AMG510 and MRTX1133) was added to each well. After 72 or 96 h, Cell Titer Glo reagent was utilised to measure cell viability at multiple time points with a 200 µL volume, and the recorded luminescence was analysed using Bio Tek Synergy™ H1. The drug concentration that impedes cell viability by 50%, termed IC50, was determined through the application of a varying scale logistic curve fitting.

For compound screening system, 1 × 10^2^ human adenocarcinoma cells (HCC827, PC9 and NCI‐H1975) co‐cultured with CM obtained from 3T3 control or Cxcl14‐OE cells for 96 h suspended in 40 µL 1640 medium were seeded to 384‐well plates. After 24 h, 92 types of drugs were prepared at their *C*_max_ concentrations and added to the corresponding wells in 10 µL of medium. After 72 h, Cell Titer Glo reagent was utilised to measure cell viability at multiple time points with a 50 µL volume, and the recorded luminescence was analysed using Bio Tek Synergy™ H1. For synergic effect assays, cells seeded to 96‐well plates and treated with drugs (filgotinib, tazemetostat hydrobromide, sodium 4‐phenylbutyrate and osimertinib) in corresponding CM for 72 h and viability was measured using cell counting Kit‐8 (CCK‐8). The absorbance at 450 nm was measured using a microplate reader after an incubation period of 2–3 h, and the viability rate was calculated following the manufacturer's instructions. The Bliss synergy score was evaluated and visualised using Synergyfinder R package (v4.2.2).^71^

For colony formation survival assays, 4 × 10^3^ cells, specifically HCC827, PC9 and NCI‐H1975 cells co‐cultured with CM obtained from 3T3 control or Cxcl14‐OE cells for 96 h, were plated onto 12‐well plates and subjected to treatment with the specified drugs (afatinib, gefitinib, osimertinib and filgotinib) in corresponding CM mixed with 1640 medium (1:1). The cells were stained with. 5% crystal violet after being fixed with 4% PFA following a 10‐day period.

### 4.13 Animal experiments

Six‐week‐old male C57BL/6 mice and male nude mice (5 weeks old; BALB/c nu‐nu) were obtained from Jiangsu Huachuang Sino Pharma Tech Co., Ltd.^72^ for all animal experiments. The mice were housed in pathogen‐free facilities, with each sex housed separately in cages maintained at a temperature of 23 ± 3°C and humidity ranging from 40% to 70%. The lighting schedule followed a 12‐/12‐h daylight/darkness cycle, starting at 07:00.

For subcutaneous tumour models, mice were subcutaneously injected with 1 × 10^6^ LLC cells and 5 × 10^5^ Cxcl14‐OE or control fibroblasts, 5 × 10^6^ HCC827 cells and 2.5 × 10^6^ Cxcl14‐OE or control fibroblasts. 5 × 10^6^ PC9 cells and 2.5 × 10^6^ Cxcl14‐OE or control fibroblasts were co‐injected. When the tumour volume reaches 70 mm^3^, we divide mice into the following four groups: (1) the control fibroblast group, no treatment; (2) the Cxcl14‐OE fibroblast group, no treatment; (3) the control fibroblast group treated with gefitinib at 20 mg/kg, administered by gavage for 12 days; (4) the Cxcl14‐OE fibroblast group treated with gefitinib at 20 mg/kg, administered by gavage for 12 days. Similarly, 5 × 10^6^ H1975 cells and 2.5 × 10^6^ Cxcl14‐OE or control fibroblasts were suspended in PBS and injected subcutaneously into each mouse. When the tumour volume reaches 70 mm^3^, mice bearing tumours were randomly assigned to six groups (each group with six mice). (1) the control fibroblast group, no treatment; (2) the Cxcl14‐OE fibroblast group, no treatment; (3) the control fibroblast group treated with osimertinib at 10 mg/kg, administered by gavage for 12 days; (4) the Cxcl14‐OE fibroblast group treated with osimertinib at 10 mg/kg, administered by gavage for 12 days; (5) the control fibroblast group treated with filgotinib at 5 mg/kg, administered by gavage for 12 days; (6) the Cxcl14‐OE fibroblast group treated with osimertinib at 10 mg/kg, administered by gavage for 12 days combined with filgotinib at 5 mg/kg. Tumour size (length and width in millimetres) was measured with callipers, and tumour volume was calculated using the formula (*A* × *B* ^2^)/2, where ‘*A*’ and ‘*B*’ represent the long and short measurements, respectively. Finally, the mice were euthanised, and the subcutaneous tumours were assessed. Tumour samples were collected for photography and staining. No tumours exceeded the maximum size of 2 cm during the study, in accordance with current animal welfare guidelines.

For orthotopic xenografts, LLC cells (1 × 10^6^) with Cxcl14‐OE or control fibroblasts (5 × 10^5^) were suspended in PBS containing Matrigel (Corning) at a 1:1 ratio to prevent leakage from the lung parenchyma. The suspension was then injected into the lung parenchyma.

For metastasis models, male BALB/c nude mice (6 weeks old) received suspensions of LLC cells (5 × 10^5^) co‐cultured with CM from Cxcl14‐OE or control 3T3 cells for 72 h via tail vein injection. Mice were administered 300 µL of CM intraperitoneally daily for nearly 60 days. Bioluminescent imaging (BLI) was conducted weekly, where mice were administered a dose of 3 mg/kg D‐luciferin and subjected to bioluminescence imaging using the PerkinElmer IVIS Spectrum instrument.

### 4.14 Immunohistochemistry

The process began with fixing tissues in formalin, followed by dehydration with ethanol, embedding in paraffin and sectioning into slices that were 3 µm thick.^73^ Afterwards, the sections were stained with haematoxylin and eosin, then deparaffinised using xylene and ethanol, and rehydrated in water. For antigen retrieval, the slides were microwaved for 20 min in a sodium citrate buffer (pH 6.0). Following this step, the slides were treated with 3% hydrogen peroxide to inhibit endogenous peroxidase activity, and then washed with PBS buffer (pH 7.4). After blocking the slides with 3% BSA, primary antibodies were applied and incubated overnight at 4°C, with three subsequent washes in PBS (pH 7.4). Secondary antibodies labelled with horseradish peroxidase (HRP), corresponding to the species of the primary antibodies, were then added and incubated at room temperature for 50 min, followed by three additional rinses with PBS (pH 7.4). 3,3′‐Diaminobenzidine (DAB) staining was performed, and positive results appeared as brownish‐yellow. Haematoxylin was used to counterstain the cell nuclei. The tissue slices were dehydrated by sequential immersion in 75% alcohol for 5 min, followed by 85% alcohol for 5 min, two 5‐min immersions in anhydrous ethanol, and a 5‐min immersion in butanol. Finally, the slices were dehydrated and made transparent with a 5‐min bath in xylene, removed from xylene to dry slightly, and sealed with glue. The following primary antibodies were used:CD44 (1:300), PCNA (1:2000), CD31 (1:300). IHC sections were visualised using Grundium Ocus® microscope scanners and further processed using CaseViewer (3DHISTECH Ltd, Budapest, Hungary).

### 4.15 Data acquisition

scRNA‐seq data from four distinct lung cancer datasets were obtained from GEO (GSE131907,^19^ GSE148071^22^ and GSE123904^21^). Another scRNA‐seq data^34^ for late‐stage NSCLC under TKI‐targeted therapy were sourced from NCBI BioProject (accession code: PRJNA591860). A ST dataset from six LUAD sections obtained from GSE189487.^33^ We acquired survival data from lung bulk RNA‐seq datasets available on the TCGA through the GDC portal and GEO databases: TCGA‐LUAD,^24^ GSE10072,^25^ GSE30219,^26^ GSE31210,^27^ GSE32863,^28^ GSE63459,^29^ GSE68571,^30^ GSE72094.^32^

To validate the prediction of immunotherapy efficacy, four cohorts undergoing immunotherapeutic treatments were employed: GSE126044 were NSCLC patients who underwent therapy with anti‐PD‐1.^74^ Advanced NSCLC patients, pertaining to the study GSE135222, received treatment utilising anti‐PD‐1/PD‐L1.^75^ Anti‐PD‐1 immunotherapy patients with NSCLC in GSE136961.^76^ For research GSE93157, patients suffering from melanoma, lung cancer and head and neck received anti‐PD1 treatment.^77^The breast cancer chemotherapy cohort is derived from GSE14814,^29^ while the NSCLC chemotherapy cohort is derived from GSE25055.^78^

### 4.16 Sample acquisition and processing

We collected samples of pathologically diagnosed LUAD from Shanghai Chest Hospital, Shanghai Jiao Tong University School of Medicine. One sample (LUNG_A35T) from one patient was resected and immediately transferred for ST sequencing. The clinical information for this sample is provided in Table S6.

### 4.17 Spatial transcriptomics data analysis

Freshly collected lung tumour tissues from Shanghai Chest Hospital were segmented into appropriately sized tissue blocks. Surface residue was removed using lint‐free paper, followed by embedding in optimal cutting temperature (OCT) and freezing on dry ice. The embedded samples were preserved at −80°C. ST sequencing was conducted using the 10x Genomics Visium Spatial Transcriptomics platform, which is based on polyA capture mRNA. The raw sequencing reads of Visium ST sequencing data and brightfield microscopy images were processed using Space Ranger (v2.0.1). This software was utilised to detect tissue capture areas on the chip and perform alignment analysis against the reference genomes (Human: GRCh38).

The Seurat^79^ package (v4.3.0) was employed for additional data processing and quality control subsequent to the initial quality check results obtained from Space Ranger. The A35 section was integrated with six other sections from GSE189487.^33^ The sctransform function^80^ was applied for data normalisation, high variance features were identified, and consequently, the data were accommodated in an “SCT” matrix. We identified the top 3000 highly variable genes (HVGs) using the FindVariableGenes function from the Seurat package, and their expression profiles were subsequently analysed using principal component analysis (PCA) with the first 30 PCs. The results were visualised in a two‐dimensional plot using a non‐linear dimensionality reduction technique called UMAP. To perform signature scoring based on scRNA‐seq signatures, the AddModuleScore function in Seurat was applied. The SpatialFeaturePlot function in Seurat was utilised to create plots for spatial feature expression.

### 4.18 Single‐cell data preprocessing

Fastq format sequences from high‐throughput sequencing were processed using Cell Ranger (v7.0.1) for quality assessment and alignment to the reference genome (Human: GRCh38). The analysis of the data was carried out using the Seurat R package (version 4.3.0).^79^ In a quality control step, cells were flagged as low‐quality and excluded if they exhibited fewer than 300 or more than 7000 detected genes, unique molecular identifier (UMI) counts exceeding 100 000, mitochondrial gene fractions surpassing 10% or erythrocyte gene fractions exceeding 3%. Mitochondrial fractions were computed using Seurat's Percentage FeatureSet function with pattern = ‘λMT‐’. The same preprocessing pipeline was applied to all publicly available scRNA‐seq datasets used in our study.

### 4.19 Dimension reduction, clustering and annotation analysis

The gene expression matrix was normalised using the ‘NormalizeData’ function, and 3000 HVGs were identified with the ‘FindVariableFeatures’ function. To address batch effects, we employed the ‘Harmony’ function. Dimensionality reduction was achieved through PCA, and the top 15 principal components were selected using the ‘ElbowPlot’ function. Cell clustering was carried out with the ‘FindNeighbors’ and ‘FindClusters’ functions, with resolution determined by the ‘Clustree’ algorithm.^23^ Clusters were visualised in a two‐dimensional space utilising the ‘RunUMAP’ function. Characteristic genes for each cell subgroup were identified with the ‘FindAllMarkers’ function within Seurat, and cell types were assigned based on marker genes using the SingleR package (version 2.0.0) along with literature references. This analytical pipeline was also applied to all publicly available scRNA‐seq datasets incorporated in our study.

### 4.20 Cell–cell communication inference

Cell–cell interactions among different cell types were inferred using CellChat (v1.5.0).^81^ The expression matrix of cells was utilised to generate the CellChat subject. Ligand‐receptor pairs from the CellChat database, including ‘Secreted Signalling’, ‘ECM–Receptor’ and ‘Cell–Cell Contact’, were selected. Cellular communication networks were inferred through functions such as ‘computeCommunProb’, ‘computeCommunProbPathway’ and ‘aggregateNet’. To visualise the interplay between cell types, the ‘netVisual_circle’ and ‘netVisual_bubble’ functions were employed.

### 4.21 Gene set scoring

For scoring gene sets in scRNA‐seq data, we employed the UCell package (v2.3.1).^82^ Similarly, for bulk RNA‐seq data, we utilised single‐sample gene set enrichment analysis (ssGSEA) from the GSVA package (v1.46.0)^83^ to compute signature scores for each sample. Supporting information tables contain all gene sets used for signature scoring.

### 4.22 Differential‐expression analysis

To identify genes that are differentially expressed across clusters, we used the ‘FindAllMarkers’ function from Seurat (version 4.3.0) with the parameters set to logfc.threshold = .25 and only.pos = TRUE. We performed comparisons using the non‐parametric Wilcoxon rank‐sum test to calculate *p*‐values, which were then adjusted for multiple testing with Bonferroni correction across all genes. We selected the top 20 markers as signature gene sets for CAF clusters in GSE131907^19^ and PRJNA591860,^34^ detailed in Tables S2 and S7.

### 4.23 Gene ontology enrichment analysis

We conducted gene ontology (GO) enrichment analysis using the clusterProfiler package (v4.6.0).^84^ Subsequently, representative pathways were visualised using the ggplot2 package (v3.4.1).

### 4.24 Gene set enrichment analysis

We downloaded Hallmark, GO and kyoto encyclopedia of genes and genomes (KEGG) gene sets from The Molecular Signatures Database^85^ (MSigDB, http://software.broadinstitute.org/gsea/msigdb/). Subsequently, we conducted gene set enrichment analysis (GSEA) for DEGs using the clusterProfiler package (v4.6.0) and the GSEABase toolkit (v1.60.0) in R, employing default settings. Significantly enriched pathways were identified based on criteria including *p*‐value <.05, *p*.adjust <.25 and NES > 1.

### 4.25 Trajectory analysis

To investigate the differences in cellular differentiation between CAF‐C3 and CAF‐C11, we utilised the Monocle 2 algorithm. We began by applying the reduceDimension function with the DDRtree method, limiting the analysis to two components for effective dimensionality reduction. Next, we employed the differentialGeneTest function to pinpoint genes that show significant differential expression in relation to pseudotime values. To further explore gene expression patterns associated with branch fate, we implemented branch expression analysis modelling (BEAM). For visualising the expression dynamics of genes specific to each branch along the differentiation trajectories, we used the plot_genes_branched_pseudotime function. This produced curves that illustrated the Loess‐smoothed expression profiles of these genes as they varied along each trajectory.

### 4.26 Survival analysis

We conducted survival analyses to assess the clinical significance of various signatures including CAF‐C11 (see Table S2). Bulk RNA samples were processed as follows: gene expression values were converted to Fragments Per Kilobase Million (FPKM) and subjected to log‐transformation. *Z*‐scores were subsequently computed for each gene across all samples. We utilised ssGSEA from the GSVA package (v1.46.0)^83^ to compute signature scores for each sample. Subsequently, based on the cutoff values of the signature scores determined using the survminer package (v0.4.9), samples were divided into two groups. We employed the Kaplan–Meier method in survival package (v3.3‐1) to estimate survival curves for both patient groups and assessed statistical significance using the log‐rank test. We employed the multivariable Cox proportional hazard model to assess the primary factors influencing survival duration, including the relationship between signature scores and clinical characteristics (Pathological Stage: stage i = 1, stage ii = 2, stage iii = 3, stage iv = 4; Sex: ‘male’ = 1, ‘female’ = 0; Age at diagnosis: ‘>65’ = 1, ‘≤65’ = 0).

### 4.27 RNA sequencing

To evaluate the differences between Cxcl14‐OE and control 3T3/L929 cells in the transcriptome, using Trizol reagent (Invitrogen) for total RNA extraction, we carried out RNA sequencing (RNA‐seq) and sequenced the resulting libraries on an Illumina NovaSeq 6000 platform. Alignment of RNA‐seq reads to the mouse reference genome was performed using HISAT2.^86^ We employed the R package DESeq2 (v.1.34.0) to analyse the differential expression genes.^87^

### 4.28 Statistical analyses

Statistical analyses and sample sizes (*n*) for each figure are detailed in their respective legends. The data distribution was assumed to be normal, although it was not subjected to formal testing. All experiments were consistent and reproducible. Kaplan–Meier curves illustrated survival functions for both mice and humans. Statistical evaluations were undertaken with GraphPad Prism 9.0 software and R software (v4.2.0). Results are presented as mean ± standard deviation (SD), unless stated otherwise. Statistical significance was assessed using either an unpaired two‐sided Student's *t*‐test or a two‐way analysis of variance (ANOVA), with a *p*‐value of less than. 05 considered statistically significant. Significance levels are indicated with the following notation: *p < .05, **p < .01, ***p < .001, ****p < .0001.

## AUTHOR CONTRIBUTIONS

The conception and planning of the project were handled by Xufeng Pan, Yajuan Zhang, Haitang Yang and Feng Yao. Weijiao Xu was responsible for creating bioinformatics profiles, conducting all experiments and analysing the data. Weijiao Xu, Ke Xu, Baicheng Zhao, Enshuo Zhang and Anshun Zhu isolated plasma from patient blood samples. The flowchart and schematic were drawn by Yunxuan Jia and Weijiao Xu. Human LUAD samples and blood samples were supplied by Gang Liu, Jianlin Xu, Haitang Yang and Feng Yao. The manuscript was assembled by Weijiao Xu, Haitang Yang, Sean R. R. Hall and Thomas M. Marti. Ren‐Wang Peng, Yongliang Niu and Patrick Dorn provided valuable suggestions for our manuscript.

## CONFLICT OF INTEREST STATEMENT

The authors declare no conflicts of interest.

## ETHICS STATEMENT

The application of lung cancer tissue was sanctioned by the Institutional Review Board of the Shanghai Lung Tumor Clinical Medical Center, Shanghai Chest Hospital (Approval No.: KS(Y)23072), conforming to all pertinent ethical norms, such as the Helsinki Declaration as revised in 2013. Ahead of their participation in the research, comprehensive consent was secured from all the subjects involved. All procedures involving animals were executed in accordance with the Guide for the Care and Use of Laboratory Animals as published by the National Institutes of Health and received the approval from the Institutional Animal Care and Use Committee of Shanghai Jiao Tong University.

## Supporting information



Supporting Information

Supporting Information

Supporting Information

Supporting Information

Supporting Information
