# Supplementary figures and images for: Transitional CXCL14+ cancer‐associated fibroblasts enhance tumour metastasis and confer resistance to EGFR‐TKIs, revealing therapeutic vulnerability to filgotinib in lung adenocarcinoma
Source: Clin Transl Med. 2025 Mar 31;15(4):e70281. doi: 10.1002/ctm2.70281 (PMC11955843; doi:10.1002/ctm2.70281)

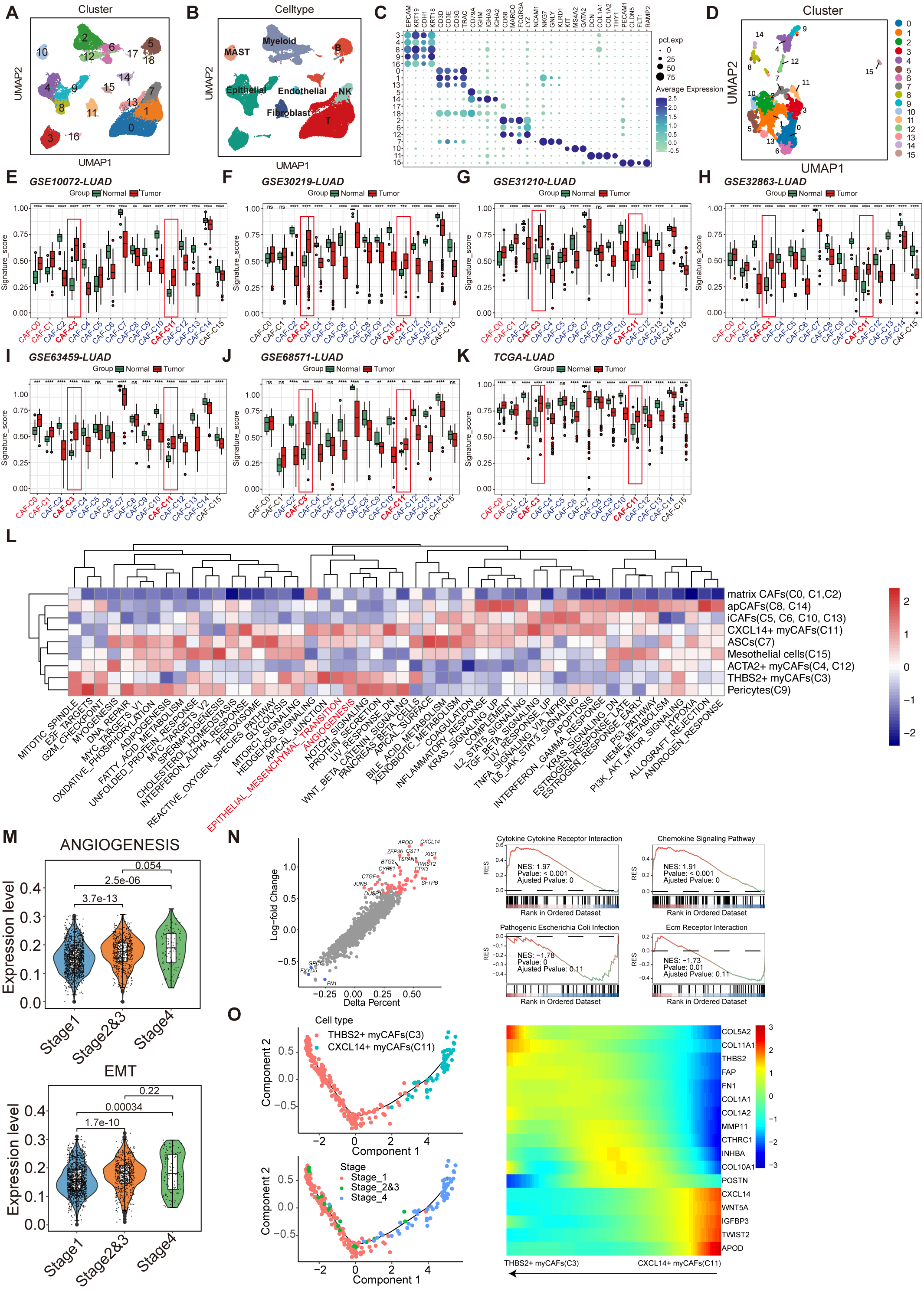

Supplement: Supplementary file 2 — Supporting Information [file CTM2-15-e70281-s004.tif]

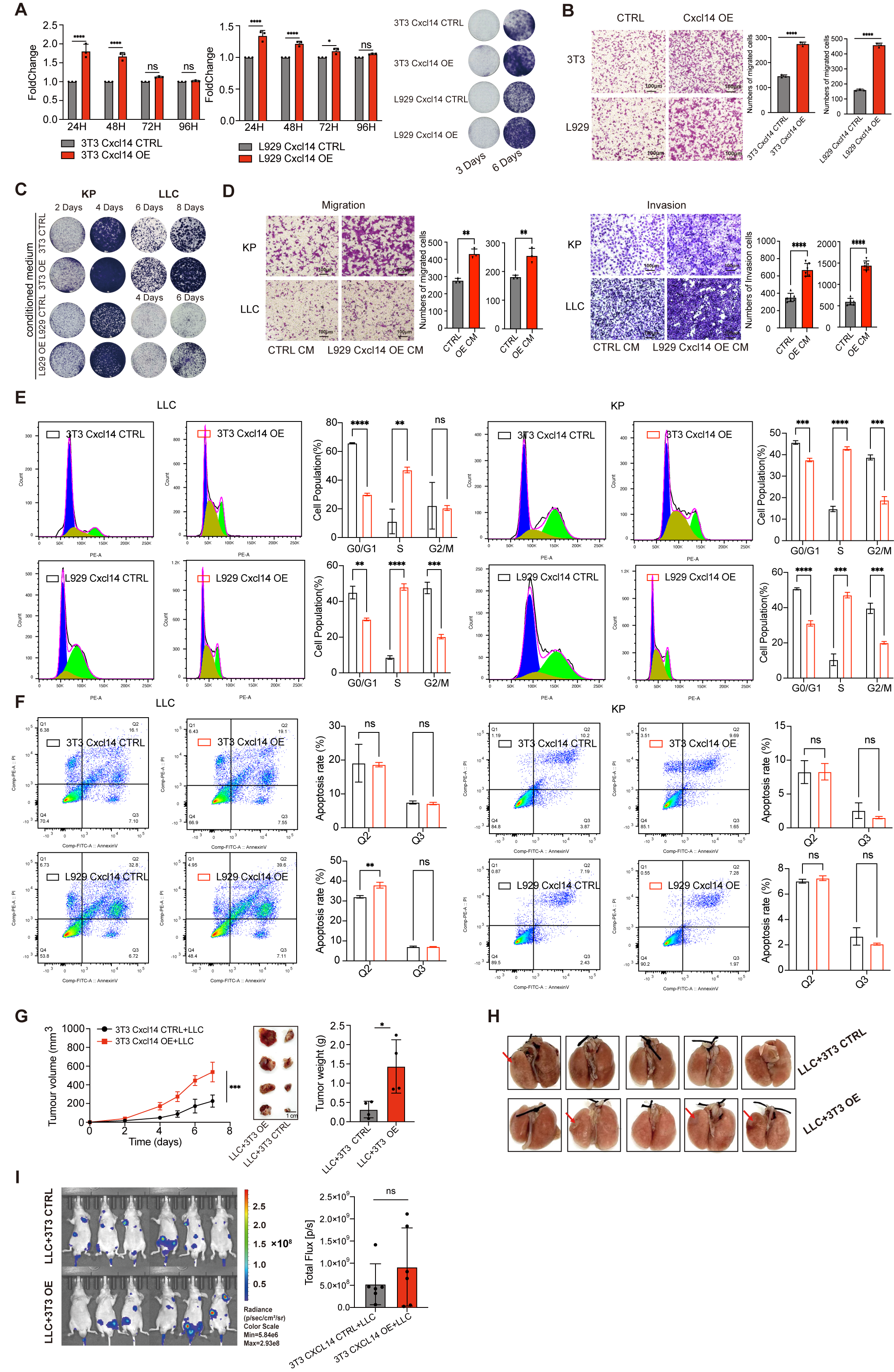

Supplement: Supplementary file 3 — Supporting Information [file CTM2-15-e70281-s001.tif]

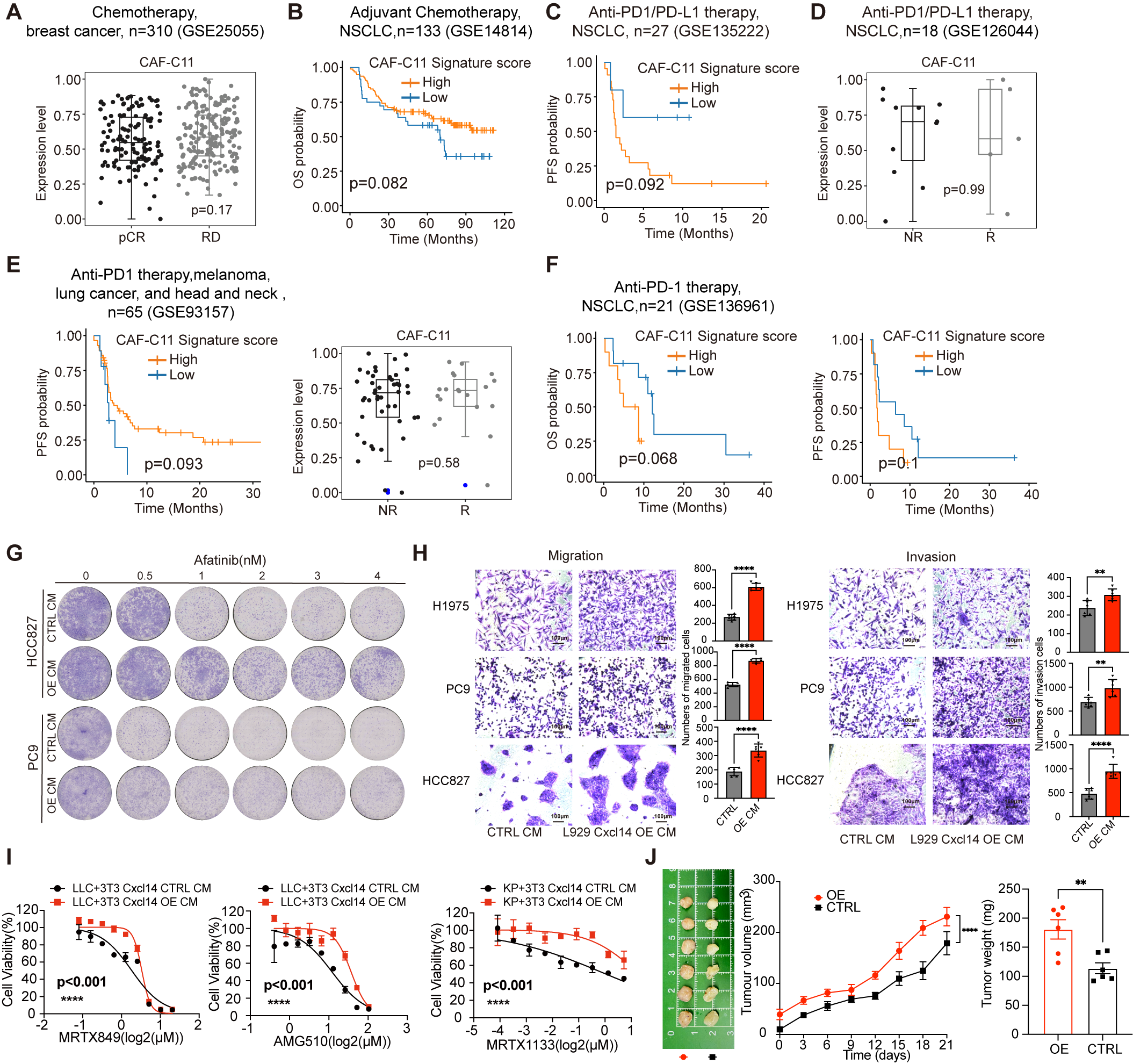

Supplement: Supplementary file 4 — Supporting Information [file CTM2-15-e70281-s002.tif]

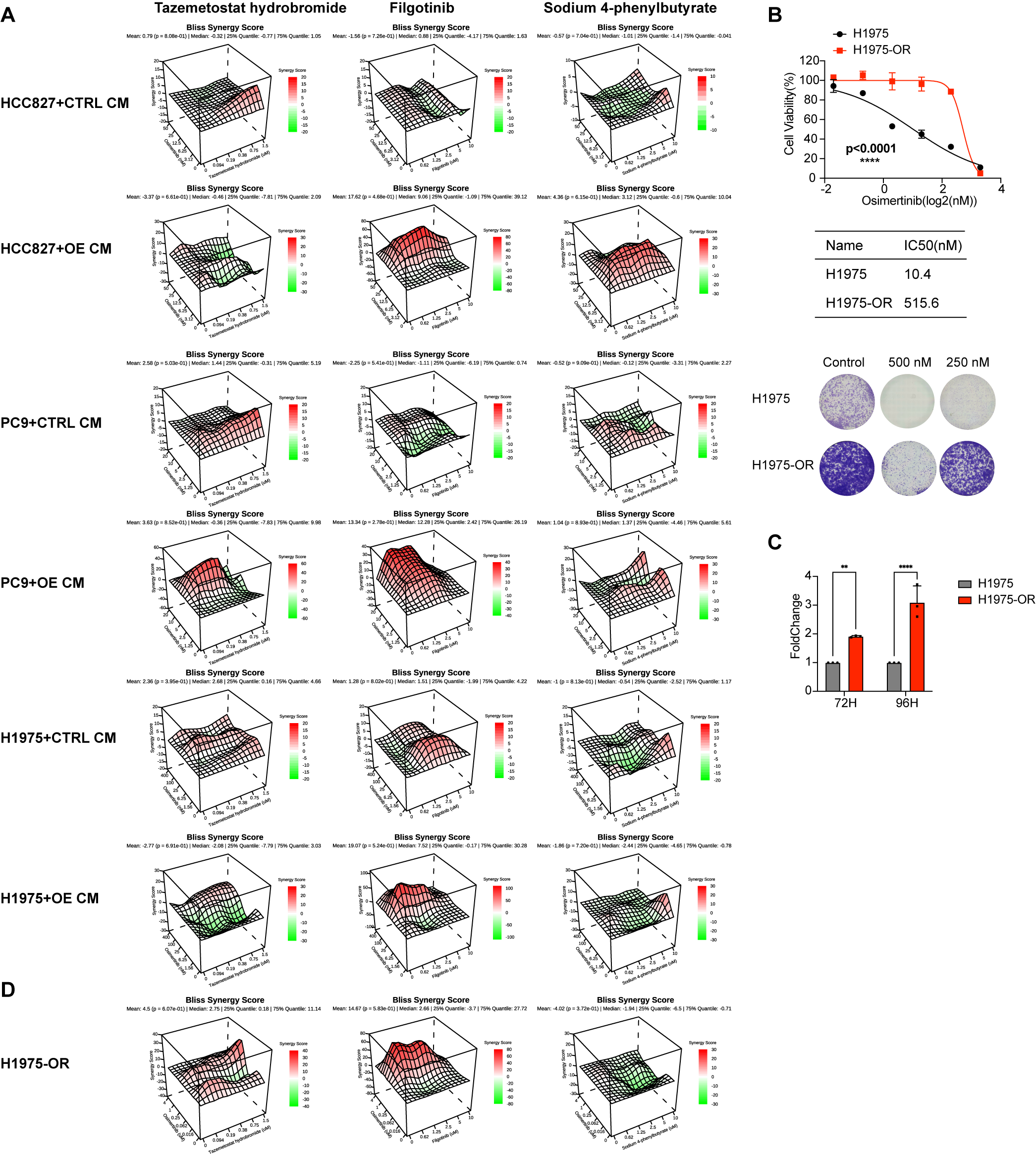

Supplement: Supplementary file 5 — Supporting Information [file CTM2-15-e70281-s003.tif]
